# Frequency-modulated timer regulates torpor–arousal cycles during hibernation in distinct small mammalian hibernators

**DOI:** 10.1038/s44323-024-00002-4

**Published:** 2024-07-02

**Authors:** Shingo Gibo, Yoshifumi Yamaguchi, Elena O. Gracheva, Sviatoslav N. Bagriantsev, Isao T. Tokuda, Gen Kurosawa

**Affiliations:** 1 RIKEN Interdisciplinary Theoretical and Mathematical Sciences Program (iTHEMS), Wako, 351-0198 Japan; 2 Institute of Low Temperature Science, Hokkaido University, Kita-19, Nishi-8, Kita-ku, Sapporo, 060-0819 Japan; 3 Inamori Research Institute for Science Fellowship (InaRIS), 620 Suiginya-cho, Shimogyo-ku, Kyoto, 600-8411 Japan; 4 Department of Cellular and Molecular Physiology, Yale University School of Medicine, New Haven, CT 06510 USA; 5 Department of Mechanical Engineering, Ritsumeikan University, Kusatsu, Shiga 525-8577 Japan

**Keywords:** Circadian rhythms, Circadian rhythms and sleep

## Abstract

Hibernation allows mammals to endure harsh seasons by reducing their basal metabolism and body temperature (Tb) to minimize energy expenditure. During hibernation in small animals such as Syrian hamsters and 13-lined ground squirrels, Tb decreases to an ambient level ( < 5 °C) and remains constant for days to weeks in a physiological condition termed deep torpor. Torpor is interrupted by periods of arousal, during which Tb recovers to a euthermic level (approximately 37 °C), and these torpor–arousal cycles are repeated multiple times during hibernation. However, little is known about the mechanisms governing Tb fluctuations during hibernation. In this study, we employed an unbiased model selection approach to Tb data and revealed that a model incorporating frequency modulation quantitatively reproduced Tb fluctuation during hibernation in Syrian hamsters. We found that an unexpectedly long period of 120–430 days modulates a shorter period of several days. In addition, the aforementioned model reproduced Tb fluctuation in 13-lined ground squirrels, which can undergo repeated hibernation according to intrinsic circannual rhythms in constant laboratory conditions. This is the first quantitative study to demonstrate the concerted action of two endogenous periods, one lasting a few days and the other lasting a year, in the torpor–arousal cycles of distinct mammalian hibernators. We anticipate that our theoretical analysis of Tb fluctuation will be a starting point for quantitative comparisons of hibernation patterns across various hibernating species. Furthermore, quantification of Tb data using models will foster our understanding of the molecular mechanisms of hibernation by revealing the biological processes operating within these periods.

## Introduction

Hibernation is a strategy allowing organisms to survive in environments with limited food and water availability^1,2^. During a season with little or no food, most small seasonal mammalian hibernators drastically decrease their basal metabolism and core body temperature (Tb) to an ambient level, often to less than 10 °C, and become immobile. This lowered metabolic and Tb state is called deep torpor (DT). DT is interrupted by interbout arousal (IBA), during which Tb rapidly increases to a euthermic level. Thus, hibernation consists of two distinct physiological states, namely DT and euthermic IBA, and Tb fluctuates between euthermia and hypothermia over intervals of a few days or weeks in a species- and temperature-dependent manner. This multiday-scale phenomenon, known as the torpor–arousal cycle, is conserved across many small mammalian hibernators^3^.

IBA is also called “periodic arousal”^4^ because of the apparent periodicity of torpor–arousal cycles. However, the length of torpor bouts gradually changes between the early and late periods of hibernation even under stable laboratory conditions, casting doubts on the periodicity of torpor–arousal cycles. Furthermore, the biological reason for these processes remains unclear, as a few species, such as tenrec and dwarf lemur, are known to hibernate without experiencing periodic arousal during hibernation^5,6^. Two mutually nonexclusive hypotheses have been proposed to explain the regulation and significance of torpor–arousal cycles^7^. First, it was suggested that the timing of arousal is regulated by proteins, protein derivatives, or metabolites during torpor^8,9^. The second hypothesis is that the timing of torpor–arousal cycles reflects circadian or circannual rhythms^10^. In nature and in human societies, some systems exhibit a gradual change in the period of oscillations^11^. For instance, the timing of sleep onset for some non-24 h individuals with sleep–wake disorder is delayed every day, and it fluctuates several times a month in a process not directly relevant to torpor^12^. This phenomenon can be understood as desynchrony between circadian rhythms and 24-h environmental cycles. However, whether this desynchronization is responsible for producing Tb patterns during hibernation has not been clarified. As recent technological advances enable Tb to be monitored for more than 100 days with high precision, quantitative analysis and phenomenological modeling of Tb time-series data can be used to address the principle governing hibernation.

The frequency change in biological time series is often quantified by the short-time Fourier transform (STFT) and wavelet transform. In the STFT, the time series, which is multiplied by a short-interval window function, is analyzed opposed to analyzing the whole time series. In wavelet transform, the basis function is localized in time and frequency, called the wavelet function, which is distinct from the trigonometric function. These two methods have been widely accepted in time series analysis. However, it is often difficult for the two methods to estimate the period of the signal within short intervals (such as analyzing the patterns of Tb fluctuation during hibernation that can be used to uncover the periodicity of torpor–arousal cycles) because of the fundamental tradeoff between time and frequency resolutions. To estimate the period of signals within short intervals, generalized harmonic analysis (GHA), a methodology usually applied to acoustics to characterize irregularities in musical or circadian rhythms, can be applied to Tb fluctuations during hibernation. In contrast to the STFT and wavelet transform, GHA can be advantageous because it simply fits the data by summing trigonometric functions based on the least squares method, and it is not bounded by the tradeoff between time and frequency resolutions.

Mammalian hibernators can be roughly classified into several categories^1,13^. One classification is based on nutrient utilization during hibernation (fat-storing hibernators and food-storing hibernators). The former category includes ground squirrels, marmots, and bears, which fast during hibernation, and the latter includes chipmunks and hamsters, which consume foods stored in their nests during the euthermic arousal phase of hibernation. Another classification is based on the timing of hibernation and torpor. Animals that can enter hibernation or daily torpor in an opportunistic manner when food or water is limited are classified as facultative or opportunistic hibernators. By contrast, obligate or “strongly” seasonal hibernators, such as ground squirrels, marmots, and bears, exhibit fall transition and repeat hibernation spontaneously even under a constant winter-like condition in the laboratory^14–20^. For example, 13-lined ground squirrels (*Ictidomys tridecemlineatus*) and Siberian chipmunks hibernate iteratively with a period of approximately 1 year under constant cold and continuous darkness^21,22^. This evidence suggests that endogenous circannual rhythms underlie hibernation in these species, which can be regarded as hibernators possessing Type II rhythms that can clock in a true circannual manner^19^. Contrarily, Syrian golden hamsters (*Mesocricetus auratus*) start hibernation in response to a winter-like short photoperiodic and cold condition in natural or laboratory conditions^4,23–26^. However, in contrast to obligate hibernators, they do not repeat hibernation with an obvious rhythm under a constant winter-like condition, leading to the notion that these species do not to have circannual rhythms, and consequently, they are categorized as facultative hibernators in many studies^24,26–29^. Nevertheless, they spontaneously exit hibernation after several months under a constant cold and photoperiodic condition, suggesting that Syrian hamsters have an unknown endogenous timer for measuring the hibernation period without any external factors^23–25^. In fact, a phenomenon called photorefractoriness has long been described in hamsters. Hamsters regress their gonads in response to chronic short photoperiods, but if the animals are housed under short photoperiod conditions for several months after gonadal regression, their gonads regrow without any environmental changes. Such photoperiod-dependent regression and photoperiod-independent regrowth led to the classification that hamsters have Type I rhythms based on an unidentified endogenous seasonal timer measuring seasonal length, similarly as strongly seasonal hibernators with Type II rhythms^30,31^. In this sense, hamsters should be classified as seasonal hibernators^32^. However, the mechanisms and principles governing such annual rhythms and seasonal timers, as well as their relationships with torpor–arousal cycles in seasonal hibernators, remain elusive.

To gain insight into the mathematical principle governing torpor–arousal cycles and hibernation, we performed a theoretical analysis of Tb across the hibernation cycle in Syrian hamsters possessing a seasonal timer and 13-lined ground squirrels exhibiting circannual rhythms^2,24,25^. Typically, the Tb time series during hibernation is noisy with irregular fluctuations, which complicates the analysis. Therefore, to uncover the temporal changes of the torpor–arousal cycle, we employed an unbiased model selection approach to Tb data obtained from Syrian hamsters and 13-lined ground squirrels. Through rigorous analysis, we unexpectedly identified a model that simultaneously reproduced the patterns of Tb fluctuation in both species. This strategic investigation both unveiled the intricacies of torpor–arousal cycles and established a robust theoretical framework, thereby advancing our understanding of the fundamental principles governing mammalian hibernation.

## Results

### Dominant period in Tb fluctuation during hibernation in Syrian hamsters changes at the 100-day scale

**Fig. 1:**
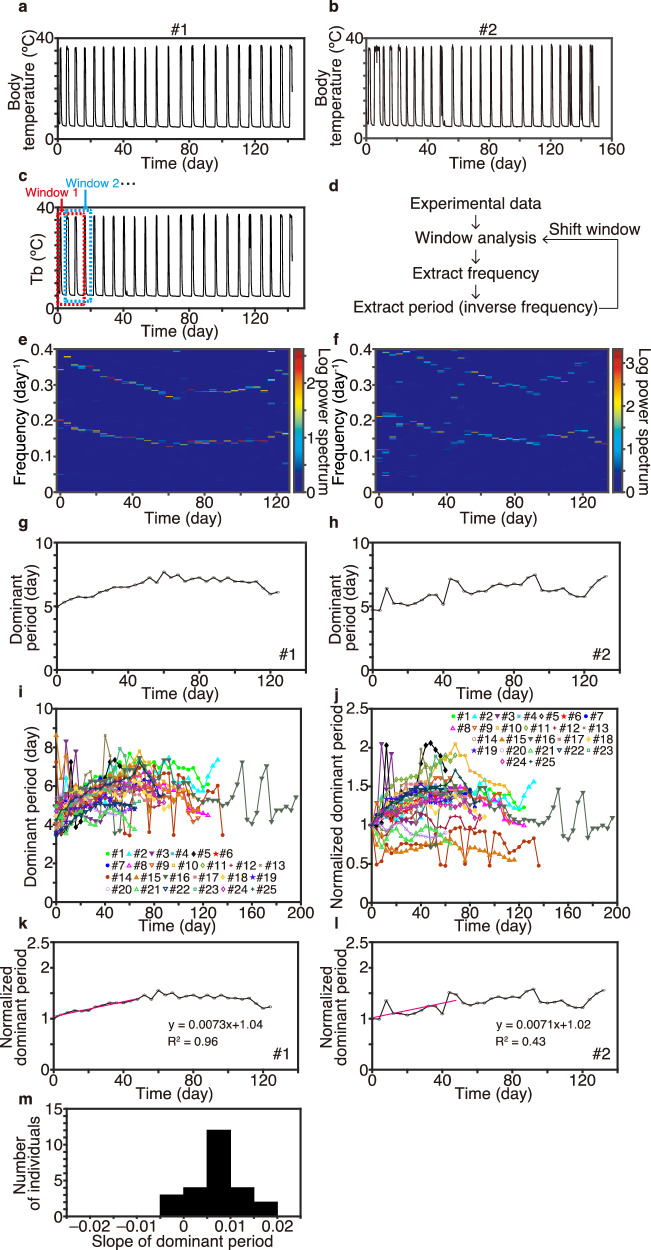
The dominant period of Tb fluctuation during hibernation in Syrian hamsters changes on a 100-day scale. **a,** **b** Time series of Tb fluctuation during hibernation under a short day and cold temperature condition (8 h light/16 h dark cycle, ambient temperature = 4 °C). Two representative hibernating Syrian hamsters (from 25 total animals) are presented in panels (**a**) and (**b**) (see also Figs. S1–S2 for data on the other animals). **c,** **d** Scheme of GHA of Tb data. **e, f** Sequence of the estimated frequency determined by analyzing data from two representative individuals during hibernation (#1, #2). The heatmaps present the magnitude of the spectrogram as the logarithmic compression of power, defined as log(1 + |amplitude|^2^). **g–i** The estimated dominant period (i.e., day/frequency) for #1 (**g**), #2 (**h**), and all 25 individual hamsters (**i**) changed over time. (**j–l**) Dominant periods normalized by the initial dominant period. **k, l** The change in the dominant period for individuals #1 and #2 at 0–48 days was quantified using linear regression. (**m**) Distribution of quantified slopes of the regression line per day for the change in the normalized dominant period.

To elucidate the rules governing Tb fluctuation during hibernation, we first applied GHA to analyze Tb in 25 hibernating Syrian hamsters^24,25^ (Fig. 1a, b, Supplementary Figs. 1–2). GHA enables accurate quantification of the periodic components underlying several torpor–arousal cycles (Fig. 1c–f) and determination of the strongest periodic component (Fig. 1g, h), which is termed the dominant frequency (“Methods”). Because torpor–arousal cycles take several days (Fig. 1a–c), we extracted the dominant frequency of torpor–arousal cycles ranging from 0.1 to 0.3 cycles per day (Fig. 1e, f), which corresponds to a dominant period of 3.3–10 days calculated as the inverse of the dominant frequency (Fig. 1g, h) to reveal the temporal changes of periodic components during hibernation. The extracted dominant periods underlying Tb time series were 3.5–8.5 days during the first 16 days after the onset of the first torpor and 4.5–8.0 days during the period of 68–84 days. GHA quantitatively revealed that the dominant period of the torpor–arousal cycle gradually changed (Fig. 1i, j). The dominant period increased or decreased over time during hibernation in individual animals (Supplementary Figs. 3–4). To compare the patterns of Tb time series, we quantified the change in the torpor–arousal cycle during the initial 48 days of hibernation using linear regression to determine whether the dominant period is proportional to time after the start of hibernation (“Methods,” Fig. 1k, l, Supplementary Figs. 3–4). These values serve to assess the relationship between the dominant period and the time after the initiation of hibernation. For 88% of the hamsters (Supplementary Figs. 3-5), the slope of the regressed line was positive with an average of 0.0068 per day, indicating that the duration of the torpor–arousal cycle increased by 0.68% per day on average over time during the initial 48 days (Fig. 1m). In particular, for 20% of the hamsters, which hibernated for more than 120 days (#1, 2, #8–10), the dominant period of the torpor–arousal cycle initially increased before decreasing toward the end of hibernation (Fig. 1g–l, Supplementary Fig. 3i, l–n, p, q). Meanwhile, 3 of 25 individuals (#3, #15, and #20) exhibited a strongly fluctuating Tb pattern, and their linear regression slopes were negative. This finding was attributable to the occurrence of shallow/daily torpor and long IBA during the first 20 days of hibernation because the dominant period of the torpor–IBA cycle tended to increase thereafter (see Supplementary Figs. 1a; 2b, m; 3a, d; 4b, e, m, p). Taken together, this analysis demonstrated that the duration of the torpor–IBA cycle changes at a 100-day scale, suggesting that it is possibly governed by an unknown physiological temporal process.

### Determination of a model reproducing the pattern of torpor–arousal cycles

**Fig. 2:**
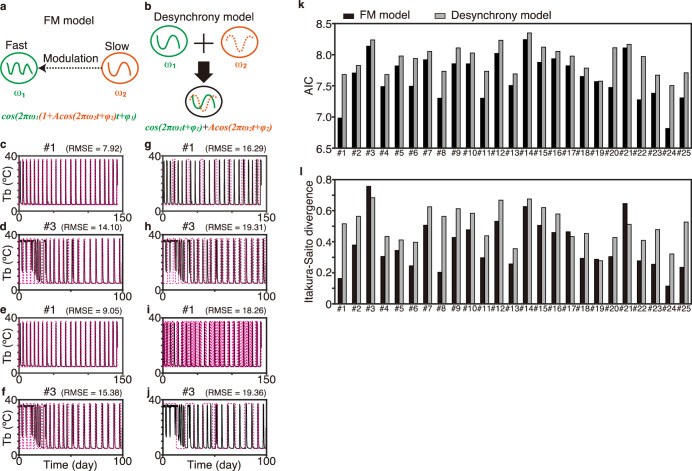
Determination of a model reproducing the pattern of torpor–arousal cycles in Syrian hamsters. **a, b** Schematic representation of the proposed FM (**a**) and desynchrony models (**b**) with multiple values of ${\omega }_{1}$ and ${\omega }_{2}$. **c–j** Comparison of simulation performed using the two models. **c–f** FM model simulation (magenta) with the best parameter set for the Tb time series recorded in two representative animals (black, #1, #3). The best parameters were chosen on the basis of the AIC (**c, d**) and IS divergence (**e, f**). RMSE, root mean square error. **g–j** Desynchrony model simulation (magenta) with the best parameter set for the Tb data from the same animals used for FM simulation (#1, #3). The best parameter was chosen on the basis of the AIC (**g, h**) or IS divergence (**i, j**). (**k, l**) Comparison of the two models for realizing each individual time series (#1–25). The likelihood values of the FM (black) and desynchrony models (gray) were compared using the AIC (**k**) and IS divergence (**l**). Note that as the model approached the experimental data, the AIC value and IS divergence decreased.

To understand the biological processes behind the gradual change in the torpor–arousal cycle, we examined the ability of two theoretical models to reproduce the experimental data, namely the frequency modulation (FM) model and the desynchrony model (Fig. 2a–l). The FM model assumes that a shorter frequency (period) might be modulated by another slower frequency (longer period). A typical example is the FM radio, which exhibits a gradual change in the period over time^33^, although few biological processes other than auditory and vocalization systems have been proposed to exhibit such features. Conversely, the desynchrony model assumes that one period, the torpor–arousal cycle period in this case, changes over time because of the failure of synchronization with another period. A typical example of this is desynchronization of the internal circadian rhythm and external photoperiod (Fig. 2a, b, “Methods”).

By varying the parameters in both models, we first identified which model better reproduced the observed Tb fluctuation using the Akaike information criterion (AIC)^34–36^. The FM model with the best parameter set, yielding the minimum AIC value, reproduced the timing of most of the transitions between DT and IBA for all individuals (Fig. 2c, d). By contrast, the desynchrony model did not reproduce most of the transitions between DT and IBA for many individuals (Fig. 2g, h). Indeed, the AIC values of the FM model with the best parameter set were always smaller for each individual’s data than those of the desynchrony model, suggesting that the FM model more accurately reproduces Tb fluctuation in Syrian hamsters during hibernation (Fig. 2k, Supplementary Figs. 6–9 in detail).

We next evaluated the agreement between the theoretical models and Tb experimental data using another statistical method, namely “Itakura–Saito (IS) divergence.” IS divergence might be a better statistical method for identifying the model and parameter set that reproduce the timing of IBA than the widely used AIC^37^ from the following points. IS divergence, originally a speech recognition method for mixed sound, features axial asymmetry, whereas the Euclidean distance has axial symmetry (see Methods and Supplementary Fig. 10). Thus, IS divergence imposes more penalties if the value of the model is smaller than the experimental data^38^. Indeed, the FM model with the best parameter set, which yielded the minimum IS divergence, reproduced most of the timings of IBA (Fig. 2e, f, Supplementary Figs. 6e, s; 11e, s). Quantification of the maximum likelihood estimation using IS divergence demonstrated that the FM model is much better than the desynchrony model for reproducing Tb fluctuation (Fig. 2l, #5, #8, and #12 in Supplementary Fig. 11). For three individuals in which the desynchrony model produced a better score than the FM model (#3, #17, and #21 in Fig. 2l), it was evident that the FM model fits the Tb data much better than the desynchrony model for most of the time series (Fig. 2f, j, Supplementary Fig. 12g, h, o, p). This suggests that judgment using IS divergence is inappropriate for 12% of individuals. Taken together, both the AIC and IS divergence statistically justified that the FM model is a proper model for reproducing periodic changes in torpor–arousal cycles.

### Two endogenous periods underlie hibernation

The aforementioned result indicates that the main property of Tb fluctuation during hibernation can be described by two key parameters in the FM model, namely shorter (day/${\omega }_{1}$) and longer (day/${\omega }_{2}$) periods. In this model, a shorter period is modulated by longer period (${\omega }_{1}$ > ${\omega }_{2}$). By determining the two periods using the theoretical model, we can quantify and explain individual variations in the complex patterns of Tb fluctuation. Because the dominant period estimated by GHA was 3.5–9 days and the longer period was approximately 100–500 days, we first rigorously varied the shorter period at the timescale of approximately 1–10 days to precisely determine the two periods (see “Methods”). To this end, we compared the estimations using the AIC and IS divergence as follows.

**Fig. 3:**
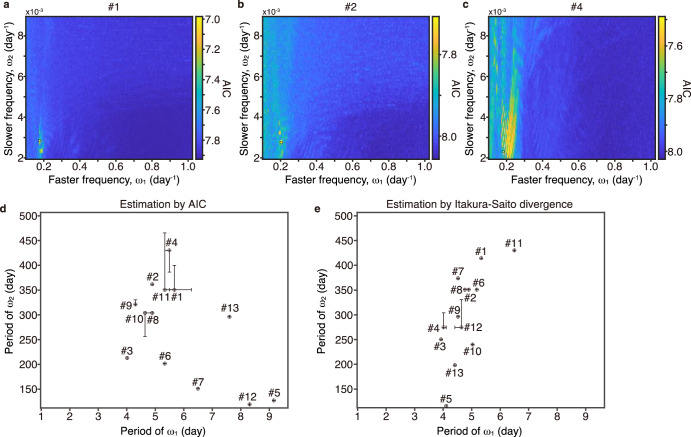
Two endogenous periods lasting several days and hundreds of days underlie hibernation in Syrian hamsters. **a**–**c** Distribution of likelihood values for the set of faster and slower periods (${\omega }_{1}$ and ${\omega }_{2}$ in the FM model) underlying Tb fluctuation was estimated using the AIC. (**d, e**) Individual variations in the set of faster and slower periods estimated using the AIC (**d**) and IS div**e**rgence (**e**). Error bars in each figure represent the range of each parameter, within which the AIC value (or IS diverg**e**nce in (**e**)) does not increase by more than 1% (0.2% in (**e**)).

Based on the minimum AIC values for the FM model that reproduced the experimental data (Fig. 3a–c, Supplementary Figs. 8–9), we determined the shorter and longer periods (${\omega }_{1}$ and ${\omega }_{2}$). The shorter period of the FM model, which yielded the minimum AIC, ranged 3.5–9.2 days, which covers the dominant period extracted by GHA (Fig. 3d). Meanwhile, the estimated longer period ranged 119–430 days (Fig. 3d). Conversely, when the parameter set of the FM model was chosen according to IS divergence, the distribution of the shorter period of the FM model (day/${\omega }_{1}$) was narrower (3.5–6.5 days, Fig. 3e). The longer period estimated by IS divergence (day/${\omega }_{2}$) spanned 115–430 days (Fig. 3e). Therefore, there was a discrepancy between the AIC and IS divergence estimations. This was probably because the FM model yielding the minimum AIC value did not reproduce the shallow/daily torpor of some individuals, whereas they were reproduced by the FM model yielding the minimum IS divergence (see #5 and #7 in Supplementary Figs. 6 and 11). Taken together, analysis using the FM model permitted the identification of a period of several days and another longer period of 115–430 days that modulates the shorter period during hibernation.

### The estimated shorter period and body weight before hibernation period are associated with the duration of hibernation

**Fig. 4:**
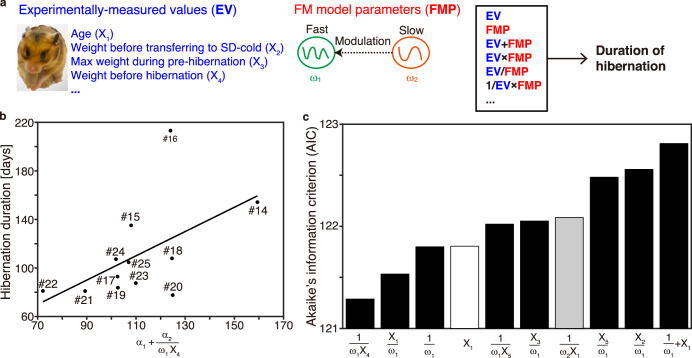
The estimated shorter period and body weight are associated with the duration of hibernation in Syrian hamsters. **a** Relationships between FM model parameters and experimentally measured physiological quantities assumed in the models. The models included age (*X*_1_), body weight before transfer to the SD-cold condition (*X*_2_), maximum body weight during the pre-hibernation period (*X*_3_), body weight before hibernation (*X*_4_), body weight after hibernation (*X*_5_), difference in body weight between before and after hibernation (*X*_6_ = *X*_5_ − *X*_4_), and estimated faster (${\omega }_{1}$) and slower (${\omega }_{2}$) frequencies in the FM model. Addition, subtraction, multiplication, and division of these values were tested. **b** The best-fit model among the 50 models chosen from the AIC to reproduce the hamster hibernation duration was that describing the duration of hibernation using the faster frequency in the FM model (${\omega }_{1}$) and the hamster weight immediately before the hibernation period (${X}_{4}$) in the form of ${\alpha }_{1}+{\alpha }_{2}/\left({\omega }_{2}{X}_{4}\right)$ with the constants ${\alpha }_{1}$ and ${\alpha }_{2}$. **c** Eight of the top 10 fitted models among 50 models tested to reproduce the hibernation duration, as chosen using the AIC, included the faster frequency in the FM model (${\omega }_{1}$), indicating that a faster frequency is important for the duration of hibernation.

The mechanisms that determine the hibernation duration remain unclear despite extensive research in various hibernators^21,39–41^. In this study, the hibernation duration in Syrian hamsters ranged from 70 to 220 days (Fig. 4a, b). We sought to determine the factors that contribute to the hibernation duration through an unknown combination (i.e., addition, subtraction, multiplication, or division) of experimentally measured physiological quantities and/or the parameters in our theoretical model. These parameters included age, body weight before transfer to the short day (SD)-cold condition, maximum body weight during the pre-hibernation period, body weight before and after the hibernation period, the difference between body weight between before and after the hibernation period (Supplementary Fig. 15), and estimated faster (${\omega }_{1}$) and slower frequencies (*ω*_2_) in the FM model. The use of the AIC enabled us to identify the best-fit model for the combination of factors that reproduced the duration of hibernation, with the number of factors in the model set as small as possible^35,36^. No single parameter in our FM model was sufficient to explain the hibernation duration (Supplementary Fig. 16). The best-fit model of 50 models chosen using the AIC (see Methods) was the combination of the faster frequency in the FM model (${\omega }_{1}$) and hamster weight immediately before the hibernation period (${X}_{4}$) in the form of ${\alpha }_{1}+{\alpha }_{2}/\left({\omega }_{1}{X}_{4}\right)$ with the constants ${\alpha }_{1}$ and ${\alpha }_{2}$ (Fig. 4a–c). This analysis demonstrated that the combination of ${\omega }_{1}$ estimated from the hibernation Tb time series and body weight immediately before the hibernation period well explained the duration of hibernation in Syrian hamsters.

### Forecasting body temperature fluctuation during hibernation

**Fig. 5:**
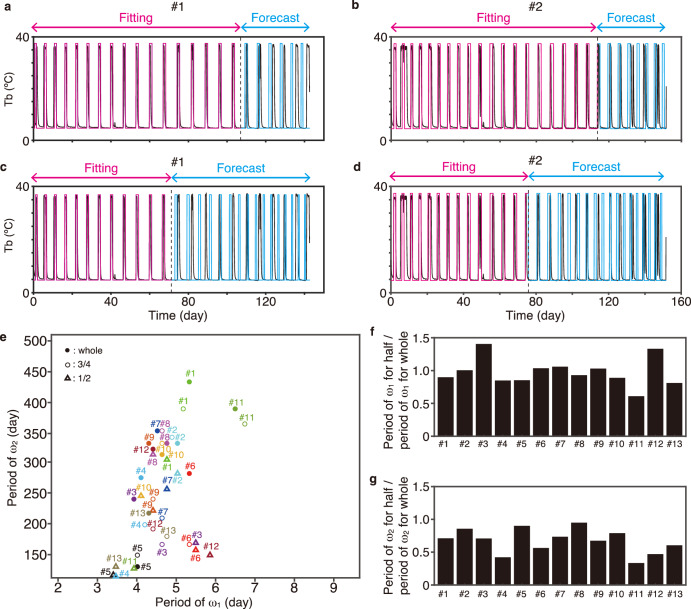
Forecasting Tb fluctuations during hibernation in Syrian hamsters. The Tb time series was reconstructed (magenta) and predicted (cyan) using three-quarters (**a,** **b**) and the first half (**c,** **d**) of the whole time series of the original Tb data in animals (black). **e** Predicted faster and slower periods (${\omega }_{1}$ and ${\omega }_{2}$ in the FM model) using partial time series based on the IS divergence estimation. **f** Ratio of the ${\omega }_{1}$ period for half of the time series to that for the whole time series. **g** Ratio of the ${\omega }_{2}$ period for half of the time series to that for the whole time series.

The validity of the FM model for Tb fluctuations can be assessed according to its predictability. To test this, we estimated two parameters (day/${\omega }_{1}$ and ${\omega }_{2}$) from three-quarters (Fig. 5a, b) of the whole time series of Tb data for each individual by the FM model using the AIC (Supplementary Figs. 17, 18) or IS divergence (Fig. 5, Supplementary Figs. 19, 20). Simulation using the aforementioned parameters predicted the last quarter of the time series with good accuracy because the estimated shorter and longer periods (day/${\omega }_{1}$ and day/${\omega }_{2}$) from three-quarters of the Tb time series were similar to those estimated from the whole time series (Fig. 5a, b). When we performed the simulation with the FM model using the parameters estimated from the first half of the whole time series of Tb data, the first few cycles of the second half of the original whole Tb time series were accurately predicted, but the prediction gradually deviated from the original (Fig. 5c, d). Indeed, the longer periods (day/${\omega }_{2}$) predicted from the first half were distinct from those estimated from the original whole Tb time series (Fig. 5e, g), implying that estimation of the parameter requires a sufficiently long experimental Tb time series. Conversely, when ${\omega }_{1}$ was predicted from the first half of the whole time series data, it ranged from 3.4 to 5.9 days, and it was similar to that estimated from the whole dataset (Fig. 5e, f). These results suggest that the estimation of ${\omega }_{2}$ is influenced by the length of the Tb time series used for statistical analysis, whereas the estimation of ${\omega }_{1}$ remains robust regardless of the length of the Tb time series.

### The estimated longer period might correspond to circannual rhythms in 13-lined ground squirrels

**Fig. 6:**
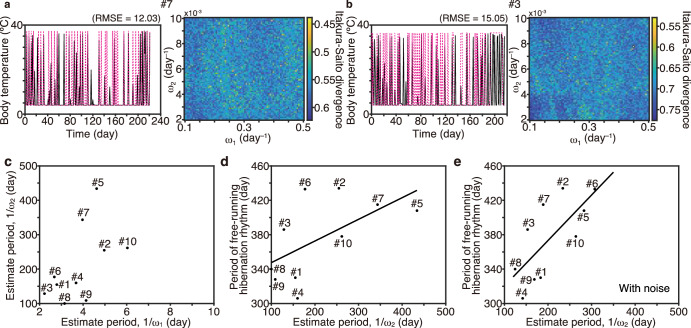
The estimated longer period might correspond to circannual rhythms in 13-lined ground squirrels. **a, b** FM model simulation (magenta) with the best parameter set for Tb time series recorded in two representative ground squirrels. **c** Individual variation in the set of faster and slower periods of the FM model, as estimated using IS divergence. **d** The measured period from the onset of hibernation to that in the next year as a function of the estimated longer period of the FM model (R^2^ = 0.3453 and p = 0.074 [*t*-test]) and (**e**) that of the FM model with Gaussian noise (R^2^ = 0.5065 and *p* = 0.021 [*t*-test]). Estimated longer period in (**e**) is the average of the best five parameter values in the noise simulation that fit the data well.

The longer period (day/${\omega }_{2}$) estimated from Tb data during hibernation in Syrian hamsters was on a scale of 100 days, in line with the seasonal rhythms of mammalian hibernators. However, although Syrian hamsters hibernate in response to winter-like condition and exit hibernation depending on the intrinsic timer within their bodies, they do not repeat hibernation in a circannual manner in a constant laboratory condition. In addition, the duration of the hamster Tb data itself was shorter than 1 year. Consequently, we cannot deduce any possible relationship between the estimated longer period (day/${\omega }_{2}$) in the FM model and circannual rhythms. To test the relationship between the longer period (day/${\omega }_{2}$) and circannual rhythms, we analyzed Tb data from 13-lined ground squirrels, a strongly seasonal hibernator that repeats hibernation over 2 years under laboratory conditions^2^, consistent with our observations (Fig. 6, see methods). The FM model yielding the minimum IS divergence reproduced most parts of Tb fluctuations for many individuals (Fig. 6a, b and Supplementary Fig. 21, 22). The shorter period of the FM model yielding the minimum IS divergence ranged 2.2–6.0 days. The longer period of the FM model estimated from the first year of the hibernation period ranged from 100 to 434 days. We then examined the relationship between the estimated longer period and the period from the onset of hibernation in the first year to that in the next year (free-running hibernation rhythm)^21^. The estimated longer period tended to be positively correlated with the period from the onset of the first hibernation period to that in the next (second) hibernation period (Fig. 6c, d, R^2^ = 0.3453, *p* = 0.074 [*t*-test])^42^. To consider the possible role of noise, which could prevent fitting to the model, we incorporated Gaussian noise into time in the FM model with the average and standard deviation of noise was set to be 0 and 2.88 h, respectively. The FM model with Gaussian noise yielded a better fit to Tb data and the correlation between the estimated longer period and the free-running hibernation rhythm (as evidenced by reduced IS divergence, Supplementary Figs. 23 and 24, Fig. 6e, R^2^ = 0.5065 *p* = 0.021 [*t*-test]). Thus, the longer period of the FM model estimated from fluctuating Tb data during hibernation could reflect a period of free-running hibernation rhythms governed by circannual rhythms in 13-lined ground squirrels.

## Discussion

Torpor–arousal cycles and circannual rhythms remain mysteries in the field of animal physiology. Several mathematical models have been proposed to reproduce Tb changes during hibernation. In the model proposed by Hampton and Andrews^10^, it was hypothesized that the metabolic rate and circannual signals affect the equilibrium of a molecular component and the input of another component into it. Using appropriate parameter settings, this model reproduces the patterns of Tb changes during hibernation for strongly seasonal hibernators. Ruf et al. ^43^ proposed a model that supposes two processes, namely an hourglass mechanism to determine the duration of torpor bouts and a threshold process oscillating in both seasonal and circadian manners. This model bears similarity to the two-process model of sleep regulation^44,45^. These models were built on a hypothetical framework, and they successfully explained certain experimental data. Conversely, the current study took a data-driven approach to identify a suitable mathematical model for reproducing Tb changes across the hibernation cycle (>50 days) in Syrian hamsters as a model seasonal hibernator. Because the data-driven approach was not built on any hypothetical framework, it might possibly derive conclusions beyond existing frameworks. Indeed, this approach also reproduced Tb changes in other strongly seasonal hibernators, namely 13-lined ground squirrels and European hamsters^46^ (Supplementary Fig. 25), suggesting that the mechanisms of torpor–arousal cycles are conserved across various hibernators.

We first applied GHA to quantify the time series of Tb fluctuation during hibernation and then identified theoretical models that reproduce this fluctuation. This study demonstrated the effectiveness of GHA for quantifying Tb fluctuation, the patterns of which are complex and varied among animals. GHA was more suitable than the STFT or Wavelet transform for exploring Tb oscillations. Through statistical analysis of the FM and desynchrony models using real Tb oscillations of hibernators, which has not been conducted to date, we found that the FM model more accurately reproduced the pattern of Tb fluctuations during hibernation in Syrian hamsters than the desynchrony model. The same finding held for 13-lined ground squirrels (Supplementary Fig. 22). Although the FM model failed to reproduce Tb fluctuations of some animals in this species, this is probably attributable to the low resolution of the Tb data (one timepoint per day). Consequently, we cannot completely reject the desynchrony model that includes circadian rhythms and other components in the Tb dataset of 13-lined ground squirrels. To the best of our knowledge, only a few studies have applied the FM model to explain biological processes, such as circadian desynchronization in rats and changes in the oscillatory frequency of brain waves in visual perception^11,47^. The FM model hypothesizes that the frequency of oscillation (${\omega }_{1}$) gradually changes over time with another longer frequency (${\omega }_{2}$). The primary conclusion drawn from the FM model was not solely the existence of two cycles but their interaction as expressed by the aforementioned equation.

Furthermore, our statistical analysis using the identified equation derived realistic values for ${\omega }_{1}$ and ${\omega }_{2}$. The derived ranges of the period in the analysis with IS divergence in Syrian hamsters were 3.9–6.5 days for ${\omega }_{1}$ and 115–430 days for ${\omega }_{2}$. Meanwhile, the derived ranges of the period in 13-lined ground squirrels were 2.2–6.0 days for ${\omega }_{1}$ and 100–434 days for ${\omega }_{2}$. It is surprising that only Tb time series data during hibernation could be used to derive such a long period such as ${\omega }_{2}$. In particular, it is of interest that ${\omega }_{2}$ ranged widely from approximately 100 to 400 days in both species because free-running hibernation rhythms vary within similar ranges among individuals in several strongly seasonal hibernators including ground squirrels, marmots, and chipmunks^22,48^. Ground squirrels kept in a constant condition exhibit changes in body weight and torpor phenotypes seasonally^15,17,22^. Additionally, recordings over several years in ground squirrels or chipmunks kept under constant photoperiods and cold temperatures demonstrated that free-running hibernation rhythms range from 5 to 16 months and from 5 to 13 months in ground squirrels and chipmunks, respectively^15,17,21,22^. Thus, the endogenous period underlying circannual phenomena such as body weight changes and hibernation could vary among individuals. ${\omega }_{2}$ might correspond to a possible endogenous period governed by such endogenous rhythms or timers. Consistent with this idea, we found a positive correlation between ${\omega }_{2}$ and free-running hibernation rhythms in 13-lined ground squirrels. Contrarily, we found no significant correlation between ${\omega }_{2}$ and the hibernation duration in Syrian hamsters (Supplementary Fig. 16). This is similar to previous findings of no correlation between the circannual period and the hibernation duration in chipmunks, a strongly seasonal hibernator^21,39^. Thus, ${\omega }_{2}$ might reflect periods governed by circannual rhythms in 13-lined ground squirrels and an unknown seasonal timer in Syrian hamsters.

Our model also proposed that ${\omega }_{2}$ affects ${\omega }_{1}$, which has a period of a few days. ${\omega }_{1}$ could be responsible for determining the duration of torpor–arousal cycles. In many small seasonal hibernators, the length of each torpor bout gradually increases in the initial phase of hibernation, reaches its peak in the middle, and then decreases at the end of the hibernation season, even under constant ambient temperature conditions^22,49–52^. Ambient and core Tb also affect the duration of each DT bout, suggesting the involvement of a temperature-sensitive process in the regulation of torpor–arousal cycles^22,53–55^. The metabolic rate measured by oxygen consumption contributes to the determination of torpor–arousal timing in golden mantled ground squirrels and garden dormice^8,54^, although the exact metabolic process responsible for the determination of torpor–arousal timing remains unclear. It should be noted that the temperature-sensitive nature of torpor–arousal cycles is apparently different from the temperature-compensation of circadian rhythms^56,57^. In fact, several studies suggested that clock genes involved in circadian rhythms have little or no contribution to determining the timing of DT–arousal cycles during hibernation^58,59^. This evidence suggests that mechanisms other than circadian rhythms governed by transcription–translation feedback loops underlie the timing of torpor onset and arousal during hibernation. ${\omega }_{1}$ might correspond to the period generated by such mechanisms. By contrast, the timing of shallow/daily torpor, an adaptive response to winter or food shortage, during which basal metabolism and Tb decrease for a shorter period of hours, is related to circadian rhythms in some species, including European hamsters, Djungarian hamsters (*Phodopus sungorus*), which exhibit seasonal shallow/daily torpor, and opportunistic heterothermic mice entering fasting-induced torpor^18,55,58–62^. Thus, although the situation remains complex and further research is needed to determine whether ${\omega }_{1}$ is sensitive to ambient temperature during hibernation, our model provides a theoretical and quantitative framework for investigating the principles behind torpor–arousal cycles.

Taken together, our nonbiased theoretical analysis identified the FM model as a simple and powerful tool for reproducing Tb fluctuation during hibernation in both Syrian hamsters possessing a seasonal timer and 13-lined ground squirrels having circannual rhythms. The model also enabled us to identify two periods that could govern hibernation and circannual events and suggested common mechanisms for torpor–arousal cycles during hibernation among distinct small mammalian hibernators. We anticipate that this study could be a starting point for quantitative comparisons of hibernation patterns across closely and distantly related hibernators exhibiting various Tb patterns. The model will also improve our understanding of the molecular mechanisms of hibernation by revealing biological processes that operate within the two periods.

## Methods

### Animal housing and Tb measurement

The animal housing and Tb measurement procedures in Syrian hamsters were performed as described previously^24^. Briefly, female Syrian hamsters were purchased from SLC, Inc. (Tokyo, Japan) and reared under long day-warm conditions (16 h light/8 h dark [lights on at 05:00–21:00] or 14 h light/10 h dark [lights on at 06:00–20:00], ambient temperature = 24 °C–25 °C) until most animals weighed more than 100–120 g. Then, the animals underwent surgery under inhalation anesthesia with 4% isoflurane (DS Pharma Animal Health, Osaka, Japan) to intraperitoneally implant core Tb loggers (iButton®, Maxim Integrated, San Jose, CA, USA, #DS1992 L-F5 or #DS1925L-F5) coated with rubber (Plasti Dip, Performix®). After 1–2 weeks of recovery, animals were transferred to SD-cold conditions (8 h light/16 h dark [lights on at 10:00–18:00], ambient temperature = 5 °C) to induce hibernation. Animals were individually housed in polypropylene cages, and the Tb of animals was measured every 90 min within an accuracy of 0.5 °C. Cage replacement was performed every 2 weeks and skipped when animals were hibernating during DT. The Tb loggers were recovered from animals after they were anesthetized for 10–15 min via an intraperitoneal injection of pentobarbital sodium (65 mg/kg) and inhalation of 4% isoflurane and sacrificed by decapitation. All animal care and experimental procedures using Syrian hamsters were performed in compliance with the Ethics Committees of the University of Tokyo (Ethical Approval no. 18-0140) and Hokkaido University (Ethical Approval no. 18-0140).

All animal procedures using ground squirrels were performed in compliance with the Office of Animal Research Support of Yale University (protocol 2021-11497). Thirteen-lined ground squirrels were housed in temperature- and humidity-controlled facilities (hibernaculum) at Yale University. During the active season (summer–fall), animals were held in a vivarium at a room temperature of 18 °C–20 °C and a photoperiod of 12 h light/12 h dark and maintained on a diet of dog food (Iams) supplemented with sunflower seeds, superworms, and fresh vegetables, with ad libitum access to water. During the hibernation season, hypothermic animals were moved to a hibernaculum with a room temperature of 2 °C –4 °C, constant darkness (except for dim red light during temperature measurements), and 40%–60% humidity. The dim red light did not affect hibernation in this species. Red light was used during body temperature measurements because squirrels cannot perceive it, whereas humans can. All squirrels were implanted with a temperature transponder (BMDS, Waterford, WI, USA). Body temperature was measured daily in each animal. All procedures with ground squirrels were performed in compliance with the Office of Animal Research Support of Yale University (protocol 2021-11497).

### Proposed theoretical models for Tb fluctuation

To simulate Tb fluctuation, we tested two models, namely the FM and desynchrony models. In the FM model, Tb during hibernation (${Tb}\left(t\right)$) is expressed by the following equation:

1 $${Tb}\left(t\right)=S\left[\cos \left(2\pi {\omega }_{1}\left(1\,+\,{A}_{2}\cos \left({2\pi \omega }_{2}t\,+\,{\phi }_{2}\right)\right)t\,+{\phi }_{1}\right)\right]+\xi \left(t\right),$$

where the frequency of oscillation (${\omega }_{1}$) is assumed to change over time with frequency (${\omega }_{2}$). In the desynchrony model, Tb is expressed as

2 $${Tb}\left(t\right)=S\left[\cos \left(2\pi {\omega }_{1}t\,+{\phi }_{1}\right)\,+{A}_{2}\cos \left({2\pi \omega }_{2}t\,+{\phi }_{2}\right)\right]+\xi \left(t\right).$$

In both models, the Gaussian additive noise term is $\xi \left(t\right)$. For simplicity, we neglected noise concerning the timing of the Tb changes. Although the desynchrony model can yield synchronous limit cycle oscillation when ${\omega }_{1}={\omega }_{2}$, it assumes that the two frequency components have a slight mismatch, which can generate long-term modulation (also called “beating”) in the dominant period (in this case, the torpor–IBA cycle period). The step function *S* was used in both models, and this function realizes sharp changes in the Tb time series. The function *S*[*x*] is defined as *S*[*x*] = the maximum value of Tb if *x* > *θ*; otherwise, *S*[*x*] = the minimum value of Tb. To identify the models and parameters that reproduce the data, all parameters were varied in a comprehensive manner (details of variation are listed in Supplementary Tables 1–5).

### Quantification of Tb fluctuation

GHA was conducted as previously described in studies of music and circadian rhythms^63,64^. GHA was developed in signal processing to quantify periodic components within a certain frequency range, the so-called “dominant period,” by estimating the best-fitted summation of trigonometric functions for a given time series^63^. The changes in the torpor–IBA period over time were quantified by (i) setting a time window, (ii) estimating the dominant torpor frequency of each time window using GHA, (iii) shifting the time window by 4 days, and (iv) repeating (ii) and (iii) until the end of the time series. The reasons for using a shift range of 4 days were as follows: [1] an insufficiently small shift would be computationally expensive and [2] a longer shift range, such as 8 or 16 days, could make it impossible to track changes in the dominant period. We confirmed that the gradual change in the dominant period was almost identical when the shift range was changed to 1, 2, 4, 8, or 16 days (Supplementary Fig. 5). In this experiment, the time window length was set at 16 days to sufficiently cover 2–3 DT–arousal cycles. In the experiment, 25 Syrian golden hamsters were individually hibernated under laboratory conditions for more than 50 days. In this analysis, the onset of hibernation was defined as the point, at which Tb decreased to less than 15 °C. The time series of Tb within the time window of 16 days for the 25 hamsters was modeled as

3 $${T}_{b}(t)=\mathop{\sum}\limits_{j=1}^{{j}_{\max }}\left\{{a}_{j}\cos \left(2\pi {f}_{j}t\right)\,+{b}_{j}\sin \left({2\pi f}_{j}t\right)\right\},$$

where *f*_*j*_ is the frequency and *a*_*j*_ and *b*_*j*_ are the amplitudes. The resolution of frequency was 1/500 per day, and *j*_max_ is the maximum number of frequency components, which was set at 4000 (i.e., *f*_*jmax*_ = 8/day). The frequency, called the dominant frequency (text), and the amplitude within each time window were quantified by repeatedly minimizing the square residuals as follows:

4 $${\int }_{0}^{L}{\left[x\left(t\right)-\mathop{\sum}\limits_{j=1}^{{j}_{\max }}\left\{{a}_{j}\cos \left(2\pi {f}_{j}t\right)+{b}_{j}\sin \left({2\pi f}_{j}t\right)\right\}\right]}^{2}{dt},$$

where *L* is the time window length. The estimated dominant period during hibernation, which is the inverse of the dominant frequency, was plotted over time (Fig. 1).

After quantifying the changes in the dominant period of the torpor–IBA cycle during hibernation, the change in the dominant period during the initial 48 days of hibernation was specifically measured by linear regression. A positive (negative) slope of the regression line indicates that the period of the torpor–IBA cycle increased (decreased) over time.

### Statistical analysis for model and parameter selection

To identify the theoretical models and parameters that reproduce the experimental Tb data during hibernation, we used the following two statistical quantities: the AIC value^34–36^ and IS divergence. These values were defined as follows:

5 $${\rm{AIC\; value}}=-\frac{N}{2}\log \left(2\pi {\sigma }^{2}\right)-\frac{1}{2{\sigma }^{2}}\mathop{\sum }\limits_{t=1}^{N}{\left(x\left(t\right)-{x}_{{model}}\left(t\right)\right)}^{2}+2v$$

and

6 $${\rm{IS\; divergence}}=\mathop{\sum }\limits_{t=1}^{N}\left(\frac{x\left(t\right)}{{x}_{{model}}\left(t\right)}-\log \left(\frac{x\left(t\right)}{{x}_{{model}}\left(t\right)}\right)-1\right),$$

where *v* is the parameter number, *x*(*t*) is the original time series, *x*_model_(*t*) is the time series of the model, *N* is the length of the time series, and ${\sigma }^{2}$ is the variance of *x*(*t*)-*x*_model_(*t*). In Figs. 2k and 3a–c and Supplementary Figs. 8, 9, 17, and 25b, the AIC value divided by the sample size for each time series data was used for normalization (i.e., AIC/*N*). An analysis using IS divergence was conducted as previously described in the field of speech recognition^37^. In fact, IS divergence was small when the model reproduced the timing of periodic arousal in the experimental data.

To identify the best model and the best parameter for each model for reproducing the experimental data, we varied the parameters as presented in Supplementary Tables 1–5. When the AIC value decreased, IS divergence decreased, and the model more accurately reproduced the experimental data. The best parameter for each model for reproducing the experimental data was identified computationally. The minimum AIC value and IS divergence of each model using the best parameter are presented for the data of each animal in Fig. 2k–l. There was a discrepancy between the AIC value and IS divergence estimation because the AIC often imposes more penalties than IS divergence if the difference between *x*(*t*) and *x*_model_(*t*) is large. The AIC assumes that the difference between *x*(*t*) and *x*_model_(*t*) follows normal a distribution, whereas IS divergence assumes that it follows an asymmetrical distribution.

To obtain the precise values of ${\omega }_{1}$ and ${\omega }_{2}$ in the FM model, the best parameter was searched within narrow ranges of ${\omega }_{1}$ and ${\omega }_{2}$ followed by a search within various parameter values (Supplementary Table 1). In the analysis of the prediction of Tb fluctuation (Fig. 5), the lower limit of ${\omega }_{1}$ was set at 0.5358/day (1.83 days) for computation costs because the likelihood of the model with a period of approximately 24 h was always small.

### Statistical analysis of hibernation duration

The AIC was used to identify physiological factors and theoretical models reproducing the duration of hibernation^34–36^. The AIC for the model ($\hat{Y}$) and hibernation duration (*Y*) was defined as follows:

7 $${AIC}=n\left(1+\log 2\pi \left(\frac{1}{n}\mathop{\sum }\limits_{i=1}^{n}{\left(\hat{Y}-Y\right)}^{2}\right)\right)+2v,$$

where *v* is the parameter number and *n* is the sample number. Hypothetical theoretical models were used as the sum or product of several candidate factors, including age at the onset of the cold condition (*X*_1_), body weight before transfer to the SD-cold condition (*X*_2_), maximum body weight during the pre-hibernation period (*X*_3_), weight before the hibernation period (*X*_4_), weight after the hibernation period (*X*_5_), weight difference between before and after the hibernation period (*X*_6_ = *X*_5_ − *X*_4_), ${\omega }_{1}$, and ${\omega }_{2}$, in the FM model.

Addition, subtraction, multiplication, and division of the aforementioned values were tested as candidate models for the hibernation duration. The models are described as follows:

8 $$\begin{array}{cc}(1)\,\hat{Y}={\alpha }_{1}+{\alpha }_{2}/{\omega }_{i} & (i=1,\,2),\end{array}$$

9 $$\begin{array}{cc}(2)\,\hat{Y}={\alpha }_{1}+{\alpha }_{2}{X}_{j} & (j=1,..6),\end{array}$$

10 $$\begin{array}{cc}(3)\,\hat{Y}={\alpha }_{1}+{\alpha }_{2}/{\omega }_{i}+{\alpha }_{2}{X}_{j} & (i=1,\,2,{j}=1,..6),\end{array}$$

11 $$\begin{array}{cc}(4)\,\hat{Y}={\alpha }_{1}+{\alpha }_{2}{X}_{j}/{\omega }_{i} & (i=1,\,2,{j}=1,..6),\end{array}$$

12 $$\begin{array}{cc}(5)\,\hat{Y}={\alpha }_{1}+{\alpha }_{2}/{\omega }_{i}{X}_{j} & (i=1,\,2,{j}=1,..6),\end{array}$$

and

13 $$\begin{array}{cc}(6)\,\hat{Y}={\alpha }_{1}+{\alpha }_{2}/{\omega }_{1}{+\alpha }_{3}/{\omega }_{2}+{\alpha }_{4}{X}_{j} & (j=1,..6).\end{array}$$

All combinations of the candidate factors yielded 50 hypothetical models.

## Supplementary information

Supplementary Information

Dataset S1

## Data Availability

Original time-series data are presented in Dataset S1. All other data are available upon reasonable request to the corresponding authors G.K. and Y.Y.

## Code availability

All codes are available in Supplementary Information.
