# Supplementary material for: Frequency-modulated timer regulates torpor–arousal cycles during hibernation in distinct small mammalian hibernators
Source: NPJ Biol Timing Sleep. 2024 Jul 2;1:3. doi: 10.1038/s44323-024-00002-4 (PMC12912452; doi:10.1038/s44323-024-00002-4)
Supplement: Supplementary file 1 — Supplementary Information [file 44323_2024_2_MOESM1_ESM.pdf]

Supplemental Information for

Frequency-modulated timer regulates torpor–arousal cycles during hibernation in distinct small mammalian hibernators

Shingo Gibo[1], Yoshifumi Yamaguchi[2,3,4]*, Elena O. Gracheva[5], Sviatoslav N. Bagriantsev[5], Isao T. Tokuda[6], and Gen Kurosawa[1, *]

[1]RIKEN Interdisciplinary Theoretical and Mathematical Sciences Program (iTHEMS); Wako, 351-0198, Japan
[2]Institute of Low Temperature Science, Hokkaido University; Kita-19, Nishi-8, Kita-ku, Sapporo, 060-0819, Japan
[3]Global Station for Biosurfaces and Drug Discovery, Global Institution for Collaborative Research and Education (GI-CoRE), Hokkaido University; Kita-12, Nishi-6, Kita-ku, Sapporo 060-0812, Japan.
[4]Inamori Research Institute for Science Fellowship (InaRIS); 620 Suiginya-cho, Shimogyo-ku, Kyoto 600-8411, Japan.
[5]Department of Cellular and Molecular Physiology, Yale University School of Medicine, New Haven, Connecticut 06510, USA.
[6]Department of Mechanical Engineering, Ritsumeikan University, Kusatsu, Shiga 525-8577, Japan.

*Correspondence: bunbun@lowtem.hokudai.ac.jp (Y.Y), g.kurosawa@riken.jp (G.K)

This PDF file includes:

Supplementary Figures 1 to 25
Supplementary Tables 1 to 5

Other supplementary materials for this manuscript include the following:

Dataset S1
Code files:
- Main_GHA.m, GHA.m: General Harmonic Analysis
- FMmodel.m: Optimal parameter search using AIC values
- desynchronymodel.m: Optimal parameter search using AIC values
- 151223_MR305.csv: Test data

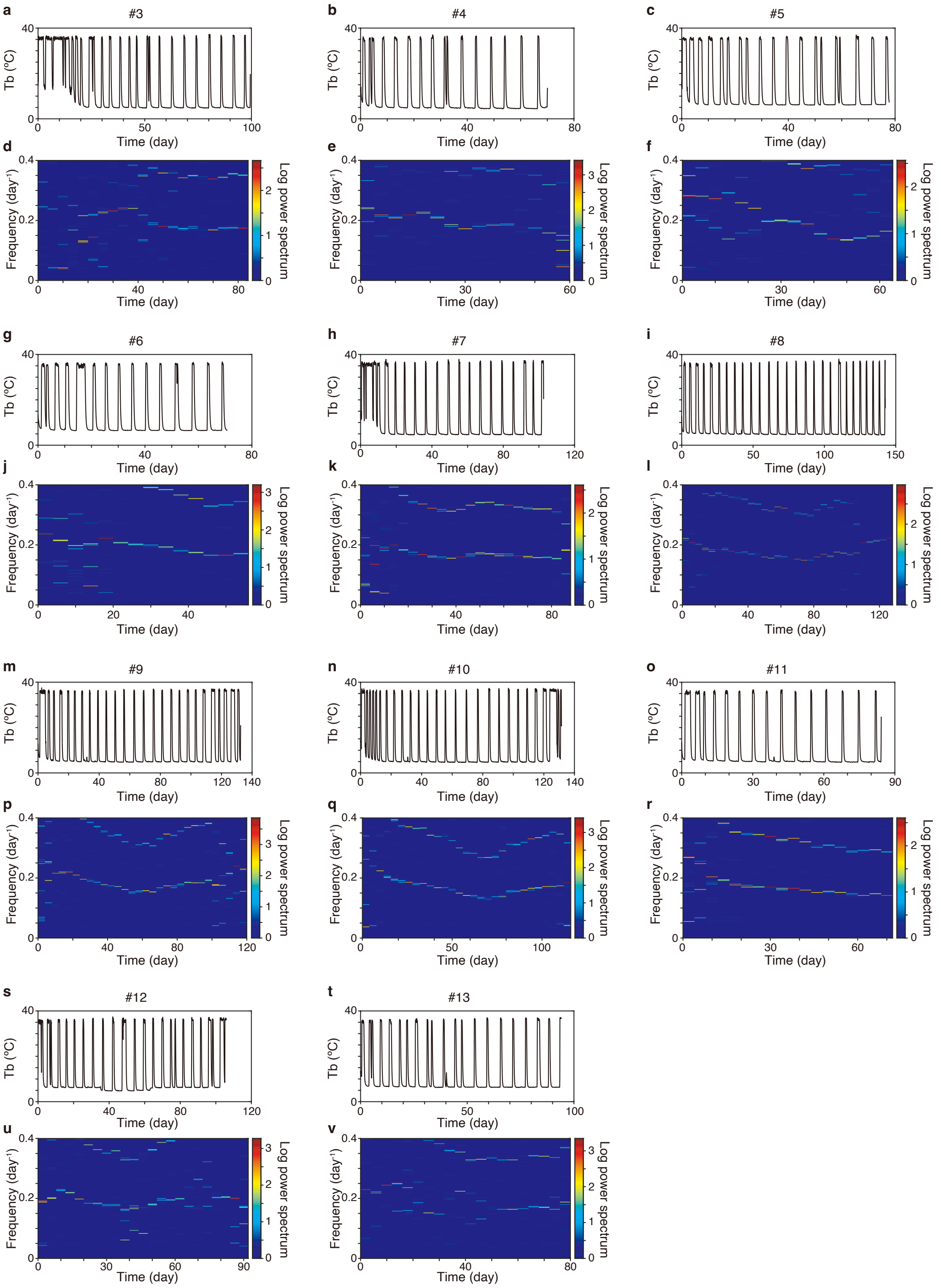

Supplementary Fig. 1: Magnitude of spectrogram by the GHA analysis of Tb data during hibernation for Syrian hamsters (a-v). Spectrogram as logarithmic compression of power ($\log(1+|\text{amplitude}|^2)$) is plotted. In our analysis, the onset of hibernation was defined as the point at which Tb was lower than 15 °C. Animal IDs (#3-13) are indicated at the top of graphs. Five of twenty-five individuals hibernated for more than 120 days (#1, 2, #8–10). A total of 25 individual datasets, including 2 from Fig. 1a, b, e, f and 12 from Supplementary Fig. 2 were analyzed.

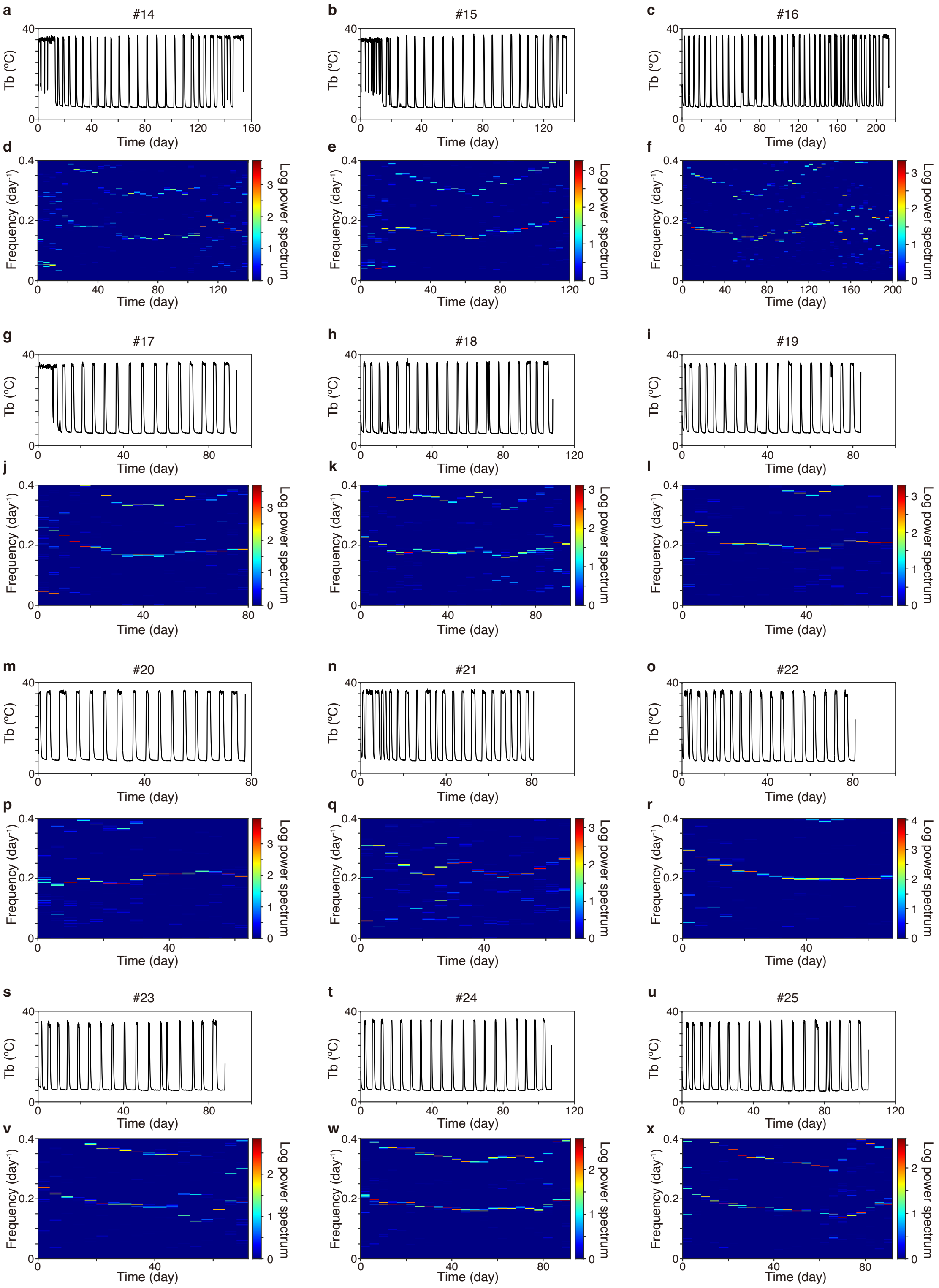

Supplementary Fig. 2: Magnitude of spectrogram by the GHA analysis of Tb data during hibernation for Syrian hamsters (a-x). Spectrogram as logarithmic compression of power (log(1+|amplitude|^2)) is plotted. In our analysis, the onset of hibernation was defined to be the point such that Tb was lower than 15 °C. Animal IDs (#14-25) are indicated at the top of graphs. A total 25 individual datasets, including 2 from Fig. 1a, b, e, f and 11 from Supplementary Fig. 1 were analyzed.

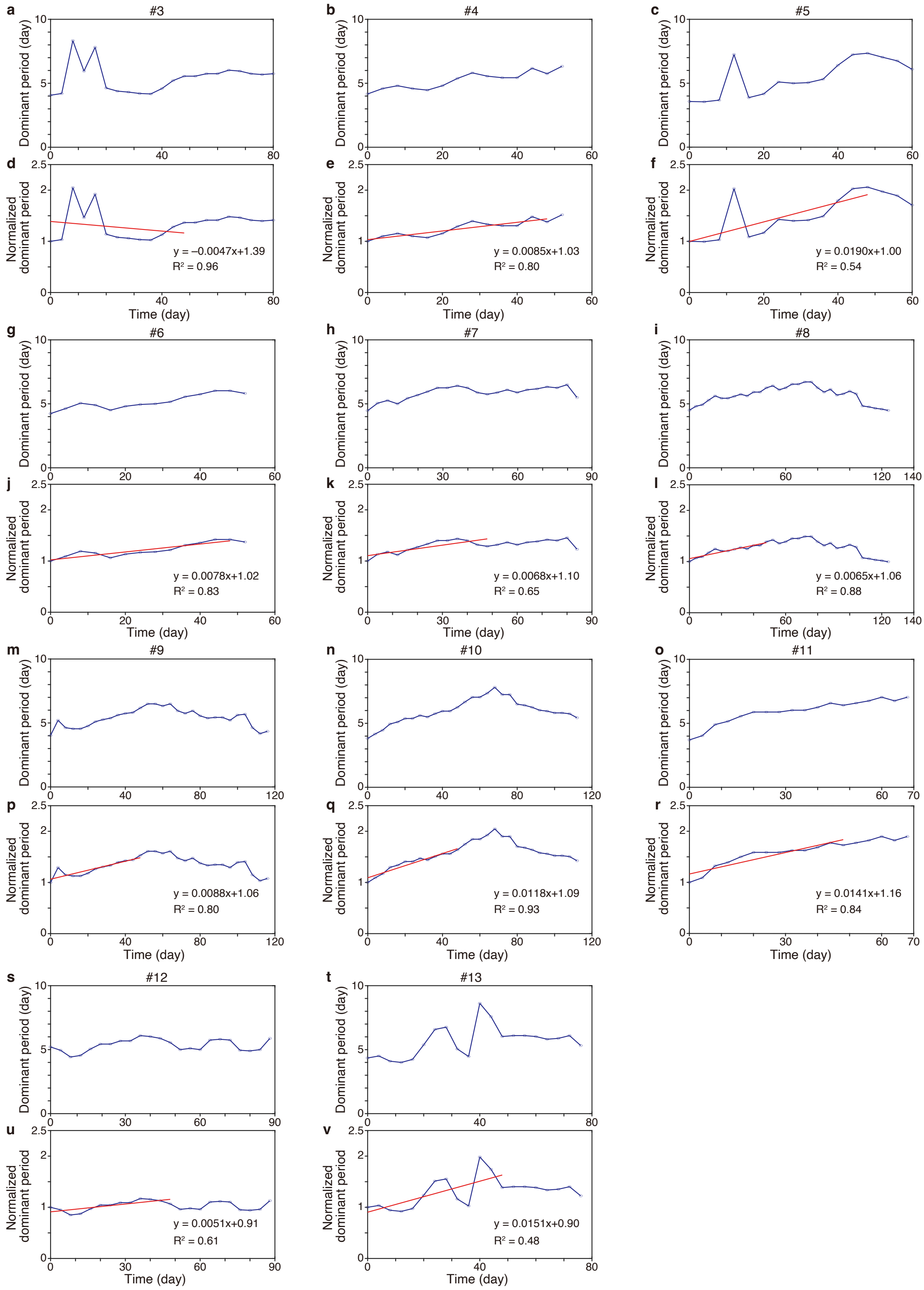

Supplementary Fig. 3: Changes in dominant period (i.e. 1/frequency) for Syrian hamsters over time, estimated by the GHA analysis (a-v). Dominant period was normalized using the initial dominant period. The red line represents the regression line for the normalized dominant period at the 0-48 days. Animal IDs (#3-13) are indicated at the top of each graph. For 22 of 25 individuals (except #3, 15, 20), the slope of regressed line was positive. A total of 25 individual datasets, including 2 from Fig. 1g, h, k, l and 12 from Supplementary Fig. 4 were analyzed.

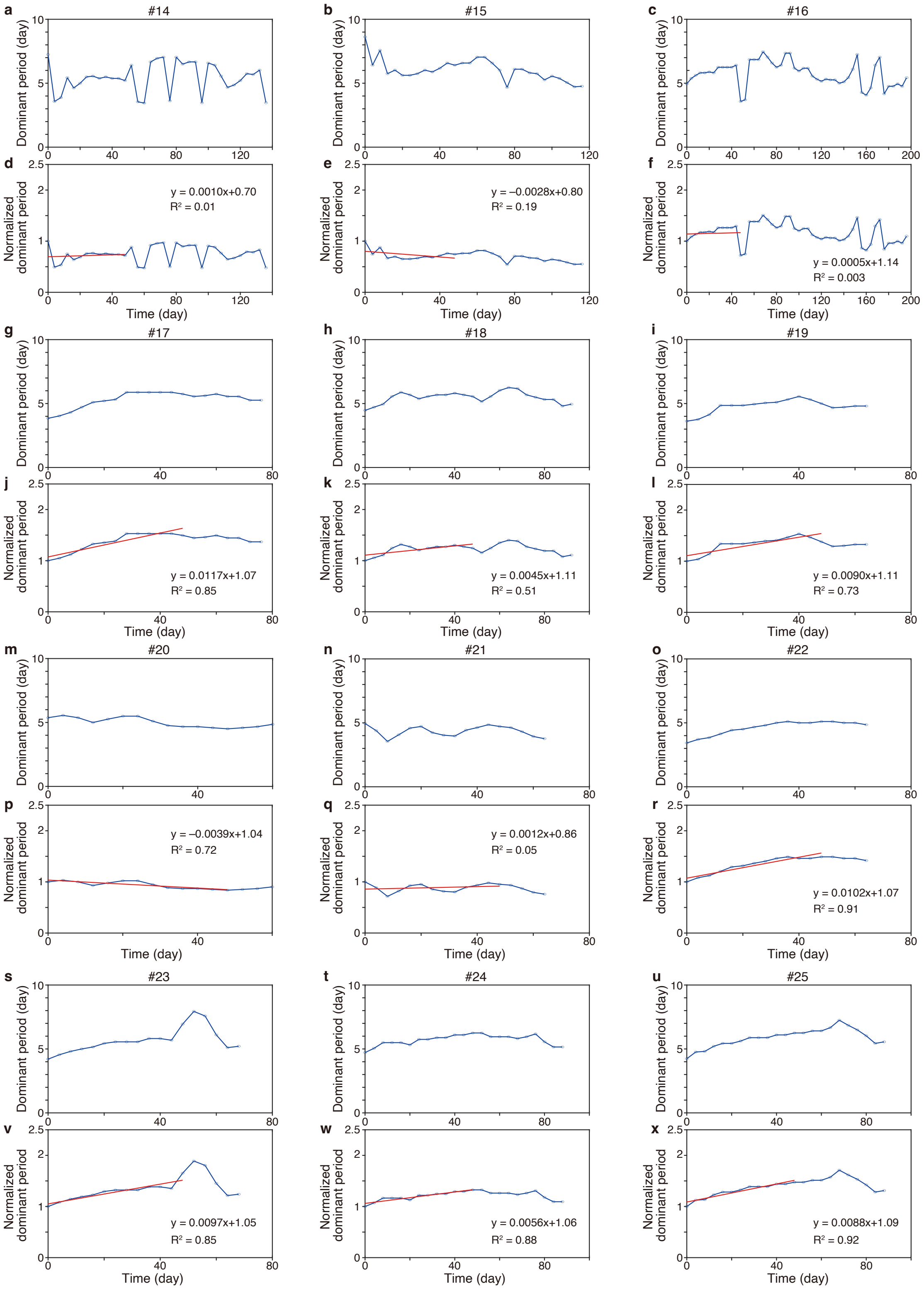

Supplementary Fig. 4: Changes in dominant period (i.e. 1/frequency) for Syrian hamsters over time, estimated by the GHA analysis (a-x). Dominant period was normalized using the initial value. The red line represents the regression line for the normalized dominant period at the 0-48 days. Animal IDs (#14-25) are indicated at the top of each graph. A total of 25 individual datasets, including 2 from Fig. 1g, h, k, l and 11 from Supplementary Fig. 3 were analyzed.

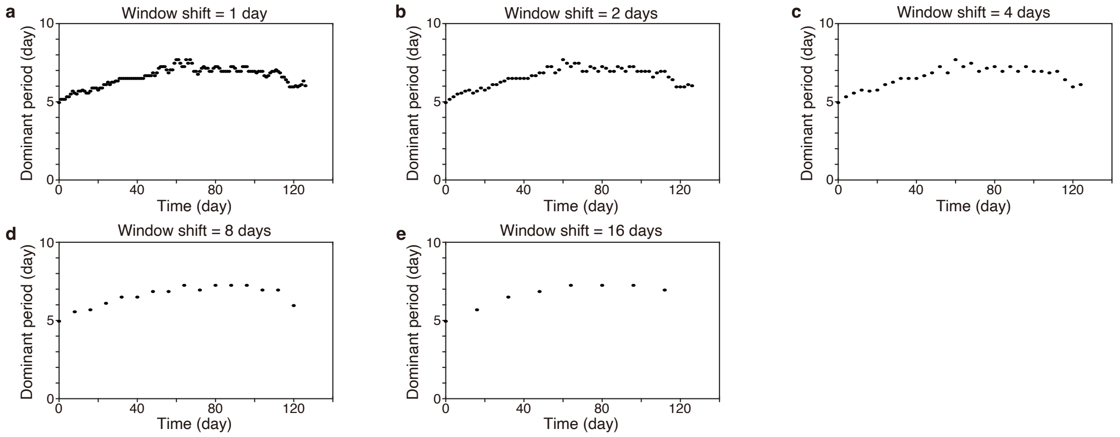

Supplementary Fig. 5: Estimated dominant period changed over time for Syrian hamster individual #1. The window shift was set to be 1, 2, 4, 8, and 16 days (a-e). A shift range of 4 days was used for GHA in Fig. 1e-l, and Supplementary Fig. 1-4.

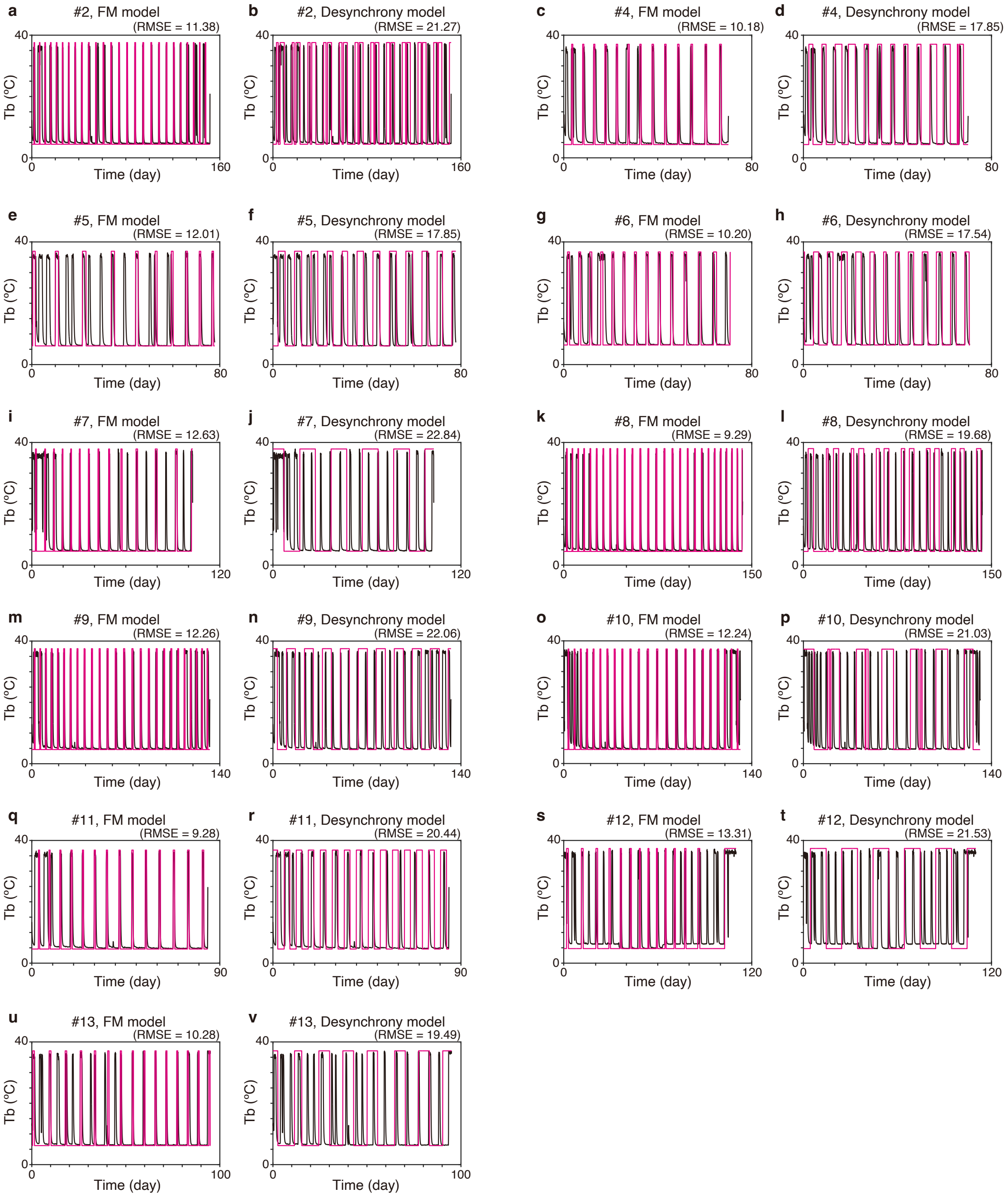

Supplementary Fig. 6: Frequency modulation (FM) and desynchrony model simulation (magenta) with the best parameter set for Tb time series in 11 Syrian hamsters (a-v). The best-fit parameter was chosen using the minimum AIC. Animal IDs (#2, 4-13) are indicated at the top of each graph. RMSE represents the root mean squared error. A total of 25 individual datasets, including 2 from Fig. 2c, d, g, h and 12 from Supplementary Fig. 7 were analyzed. Parameter ranges are shown in Supplementary Table 1 for FM model, and in Supplementary Table 3 for desynchrony model.

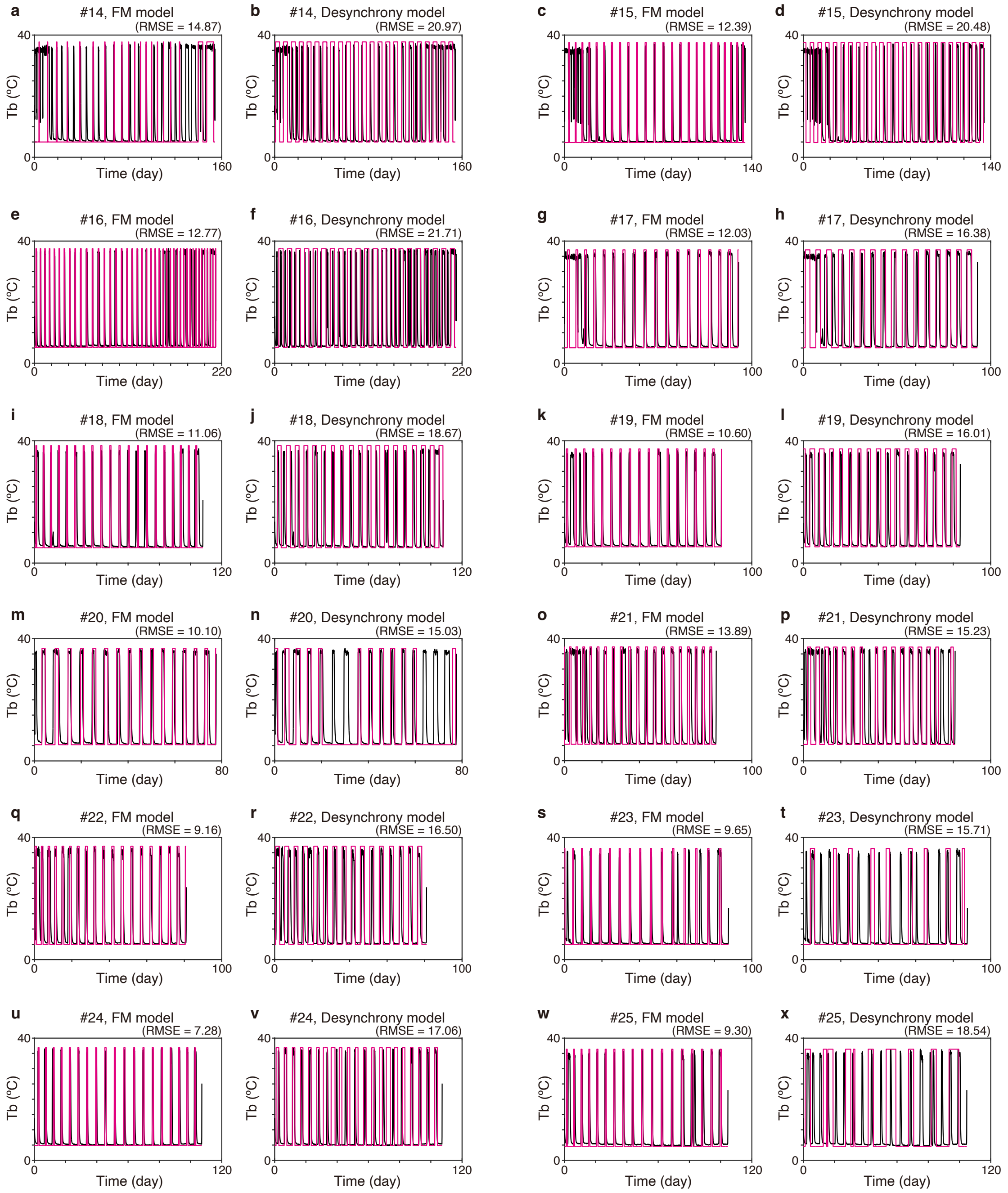

Supplementary Fig. 7: FM and desynchrony model simulation (magenta) with the best parameter set for Tb time series in 12 Syrian hamsters (a-x). The best-fit parameter was chosen using the minimum AIC. Animal IDs (#14-25) are indicated at the top of each graph. RMSE represents the root mean squared error. A total of 25 individual datasets, including 2 from Fig. 2c, d, g, h and 11 from Supplementary Fig. 6 were analyzed. Parameter ranges are shown in Supplementary Table 2 for FM model, and in Supplementary Table 3 for desynchrony model.

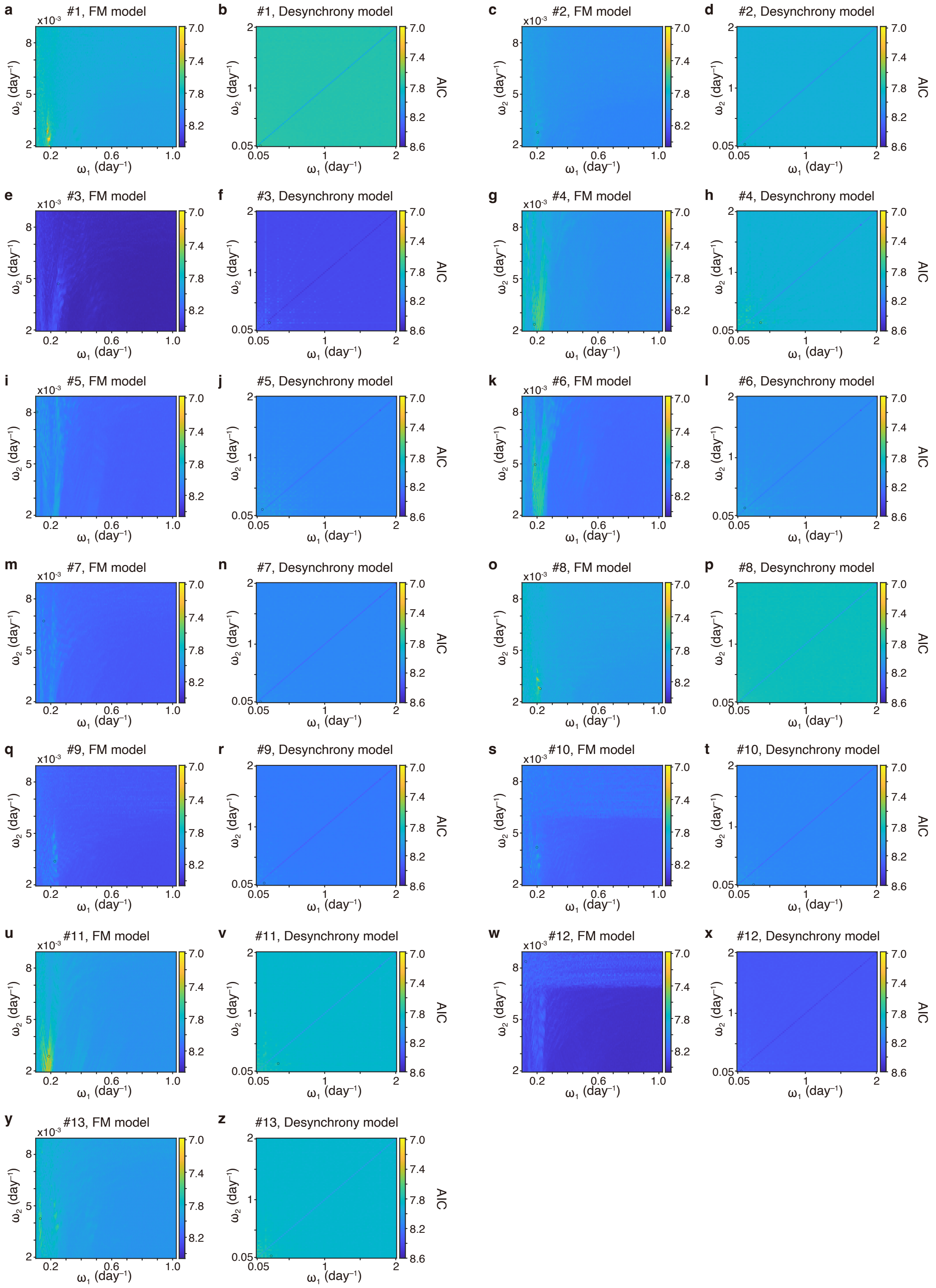

Supplementary Fig. 8: Distribution of AIC as a function of two frequencies (ω_1 and ω_2) of the FM and desynchrony models applied to experimental data from 13 Syrian hamster individuals (a-z). The best parameter set, yielding the minimum AIC (circle) was used in Fig. 2c, d, g, h, Supplementary Fig. 6. Parameter ranges are shown in Supplementary Table 1 for FM model, and in Supplementary Table 3 for desynchrony model.

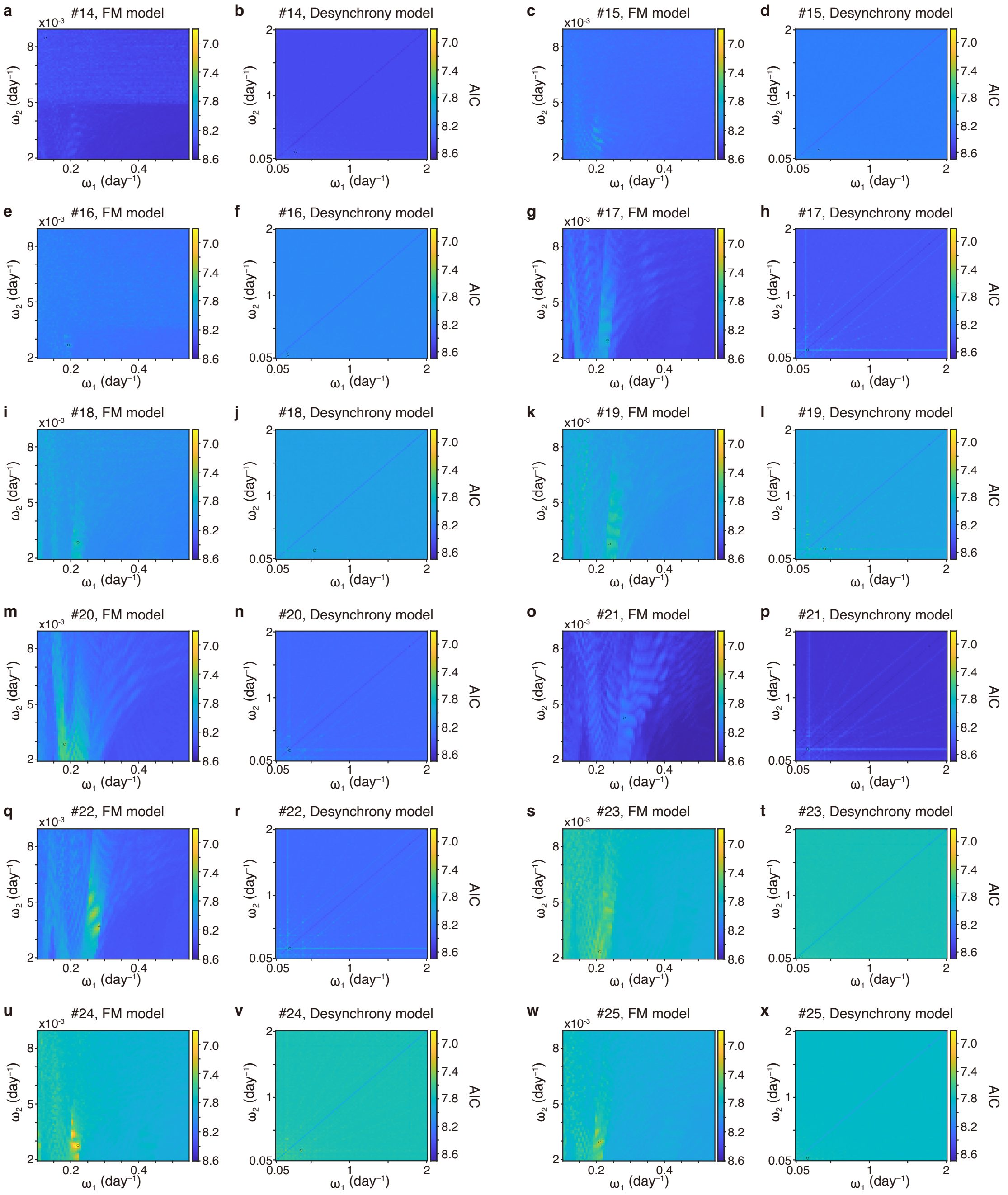

Supplementary Fig. 9: Distribution of AIC as a function of two frequencies (ω_1 and ω_2) of the FM and desynchrony models applied to experimental data from 12 Syrian hamster individuals (a-x). The best parameter set, yielding the minimum AIC (circle) was used in Supplementary Fig. 7. Parameter ranges are shown in Supplementary Table 2 for FM model, and in Supplementary Table 3 for desynchrony model.

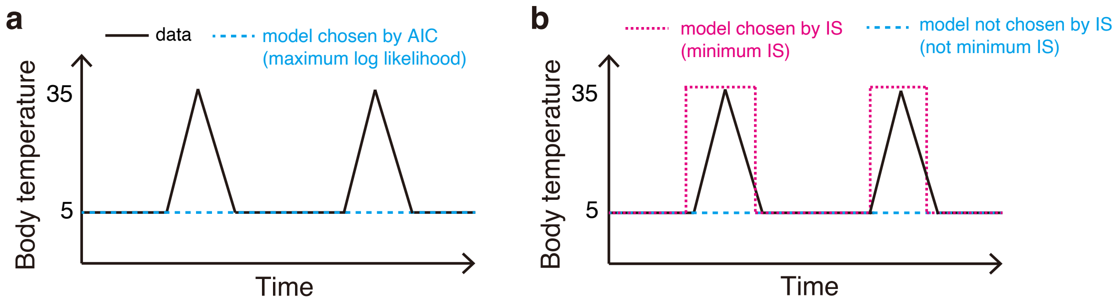

Supplementary Fig. 10: Schematic illustration of the use of AIC and IS divergence. (**a**) If the body temperature (black) is held at a certain low value for a long time and changes abruptly from time to time, the model chosen when using the AIC with log-likelihood is often one that is always held at a low value (cyan). This is because the log likelihood only calculates the logarithm of the distance between the data and the model value; therefore, the distance between the data (black) and the model (cyan) held constant is not very large. (**b**) Given the same data (black), the model chosen when using IS divergence is often one that reproduces abrupt changes (magenta). This is because the IS divergence is for data and model values of 35 and 5 is larger than that for the data and model values of 5 and 35, because the IS divergence is defined as $\left(\frac{x(t)}{x_{model}(t)} - \log\left(\frac{x(t)}{x_{model}(t)}\right) - 1\right)$ (see Methods).

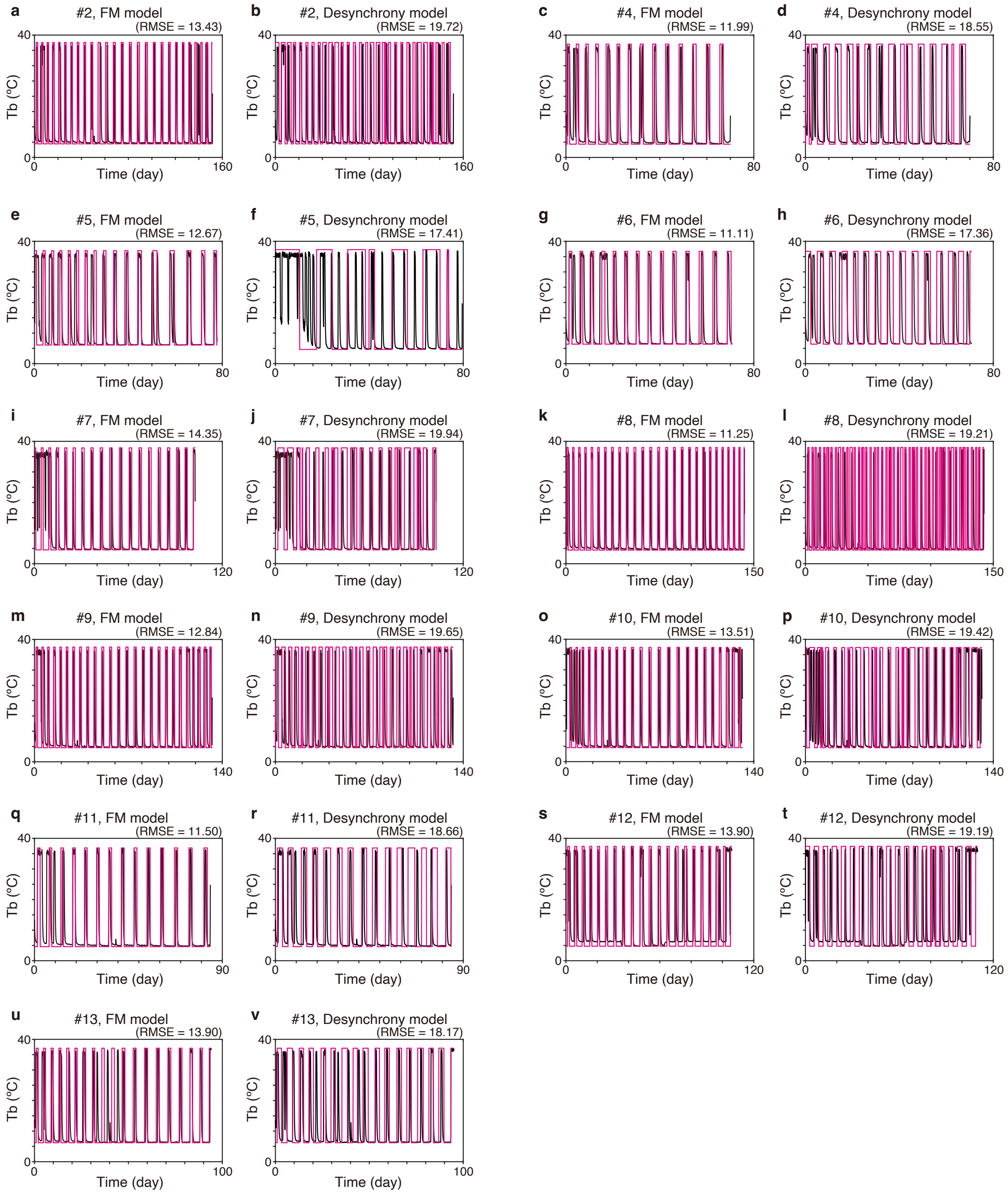

Supplementary Fig. 11: FM and desynchrony model simulation (magenta) with the best parameter set for Tb time series in 11 Syrian hamsters using the IS divergence (a-v). The best parameter set was chosen using the IS divergence. Animal IDs (#2, 4-13) are indicated at the top of each graph. RMSE represents the root mean squared error. A total of 25 individual datasets, including 2 from Fig. 2e, f, i, j and 12 from Supplementary Fig. 12 were analyzed. Parameter ranges are shown in Supplementary Table 1 for FM model, and in Supplementary Table 3 for desynchrony model.

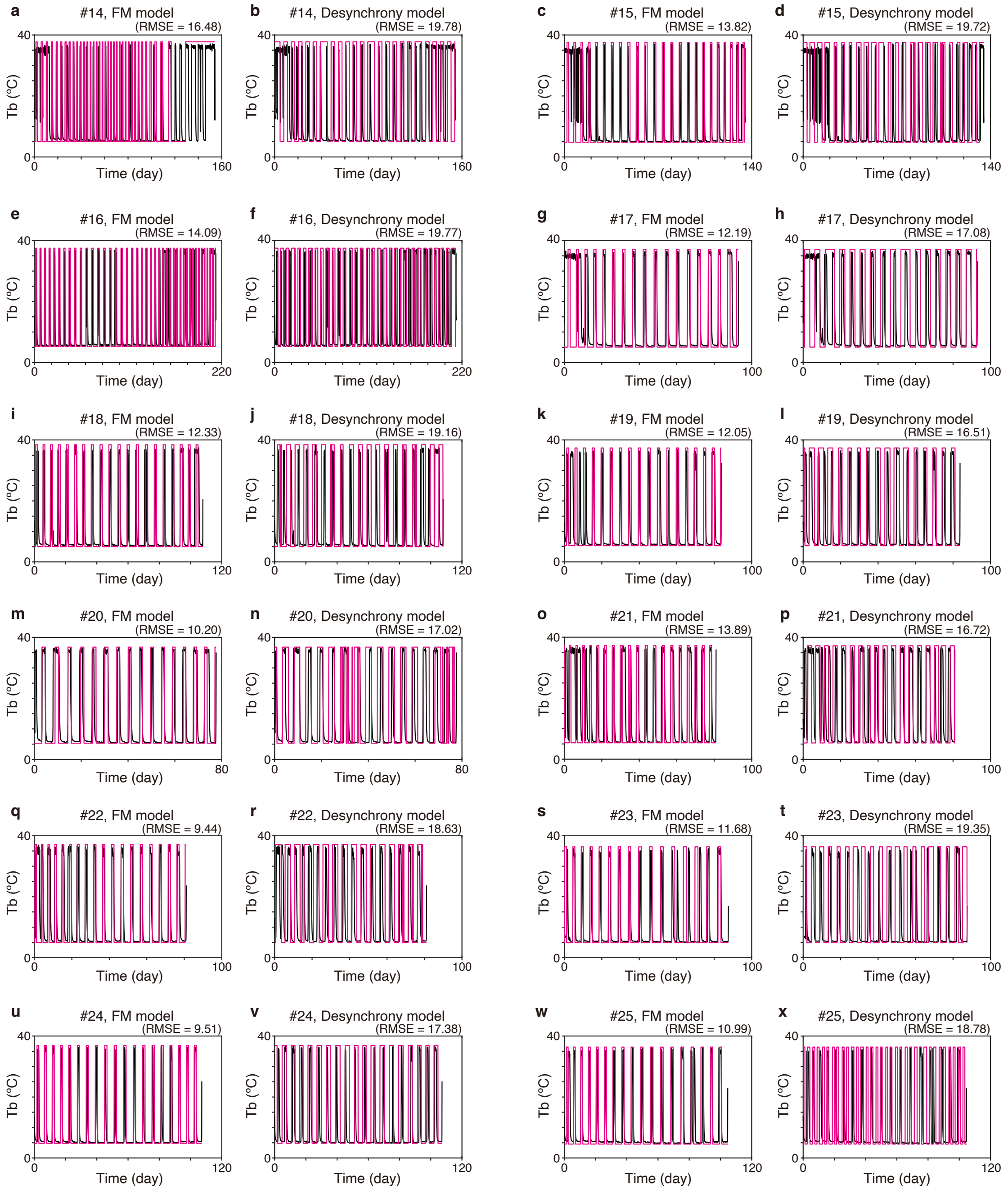

Supplementary Fig. 12: FM and desynchrony model simulation (magenta) with the best parameter set for Tb time series in 12 Syrian hamsters using the IS divergence (a-x). The best parameter set was chosen using the IS divergence. Animal IDs (#14-25) are indicated at the top of each graph. RMSE represents the root mean squared error. A total of 25 individual datasets, including 2 from Fig. 2e, f, i, j and 11 from Supplementary Fig. 11 were analyzed. Parameter ranges are shown in Supplementary Table 2 for FM model, and in Supplementary Table 3 for desynchrony model.

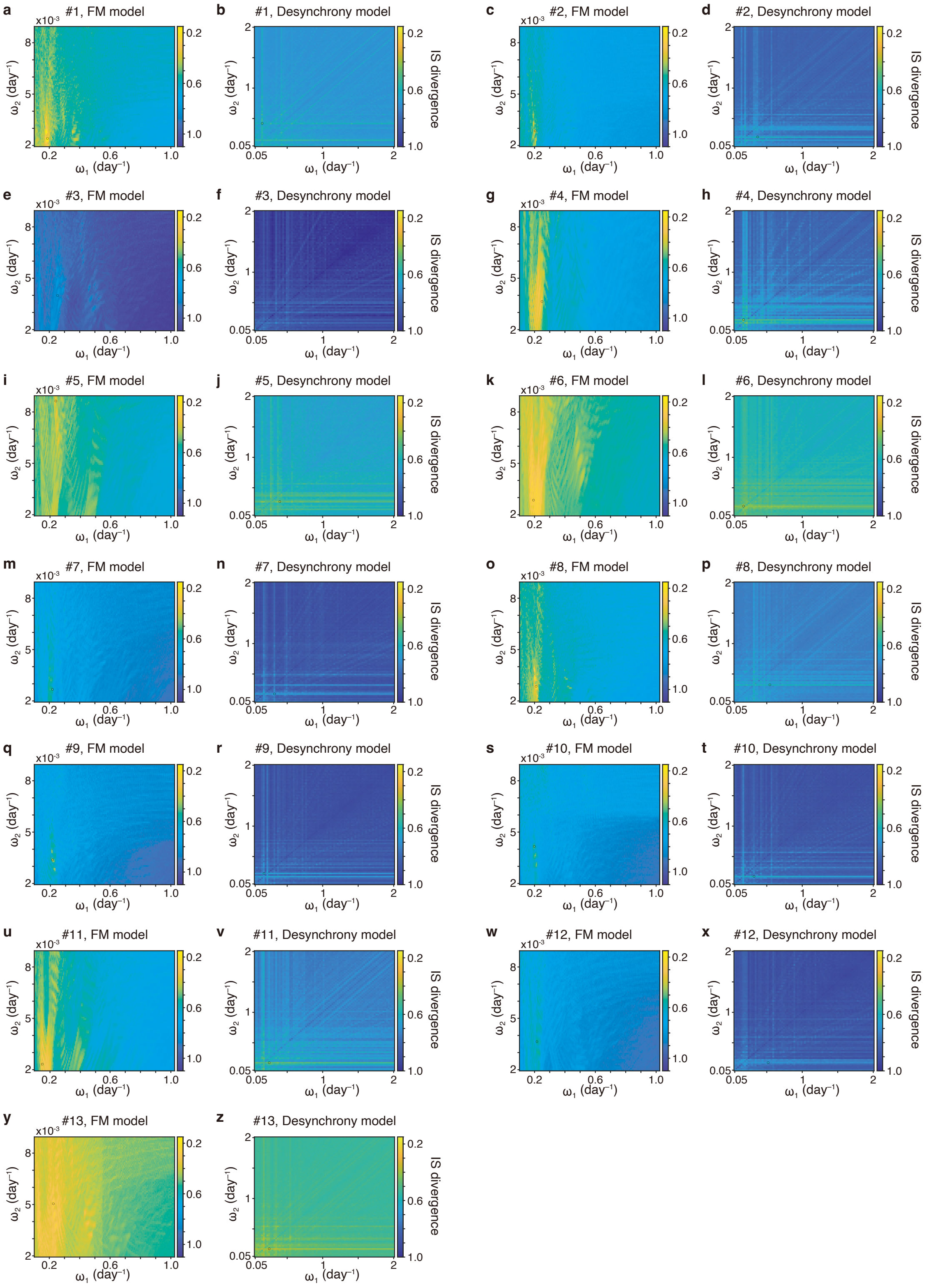

Supplementary Fig. 13: Distribution of IS divergence as a function of two frequencies (*ω*₁ and *ω*₂) of the FM and desynchrony models applied to experimental data from 13 Syrian hamster individuals (a-z). The best parameter set, yielding the minimum IS divergence (circle) was used in Fig. 2e, f, i, j, Supplementary Fig. 11. Parameter ranges are shown in Supplementary Table 1 for FM model, and in Supplementary Table 3 for desynchrony model.

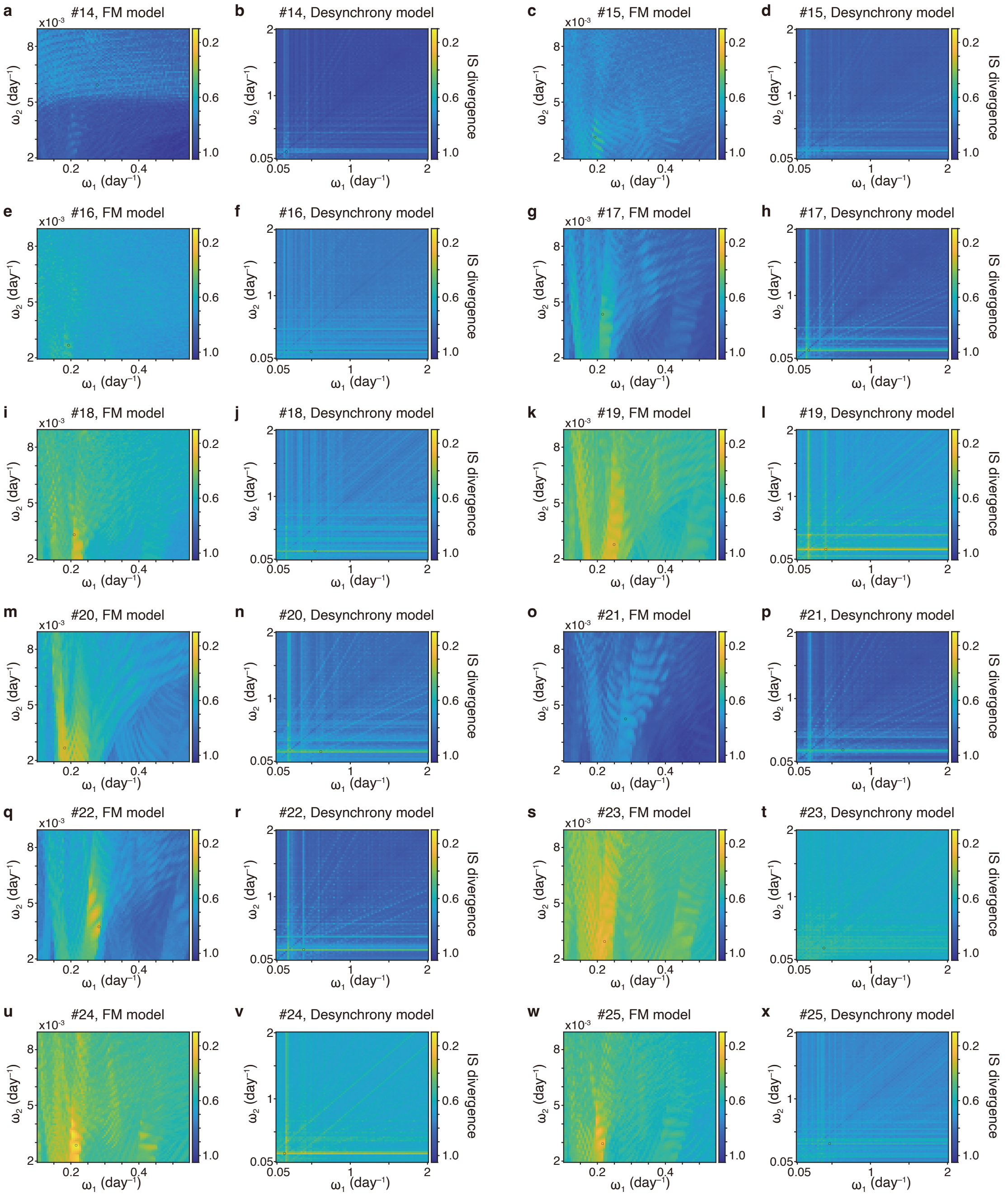

Supplementary Fig. 14: Distribution of IS divergence as a function of two frequencies (ω_1 and ω_2) of FM and desynchrony models applied to experimental data from 12 Syrian hamster individuals (a-x). The best parameter set, yielding the minimum the IS divergence (circle) was used in Supplementary Fig. 12. Parameter ranges are shown in Supplementary Table 2 for FM model, and in Supplementary Table 3 for desynchrony model.

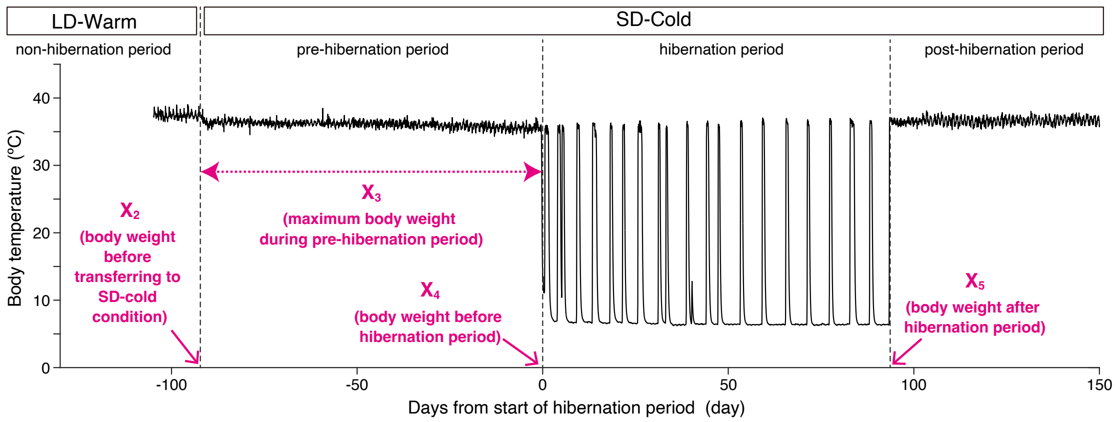

Supplementary Fig. 15: Timing of body weight measurements (X_2, X_3, X_4, and X_5) for the Syrian hamster individual used in Fig. 4.

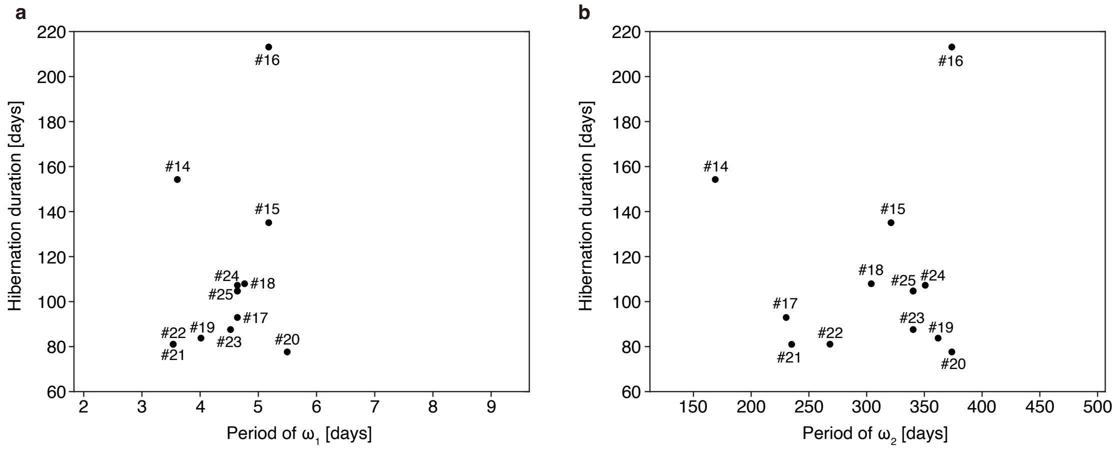

Supplementary Fig. 16: Distribution of hibernation duration as a function of faster and slower period (ω_1 and ω_2 in FM model) for 12 Syrian hamster individual experimental data (a,b). The period of ω_1 and ω_2 were estimated using IS divergence. A total of 12 individual datasets (#14-25) were quantified, for which Tb time-series of the whole hibernation period was measured. In our analysis, the offset of hibernation is defined as the point at which Tb remains higher than 15 °C for more than 10 days.

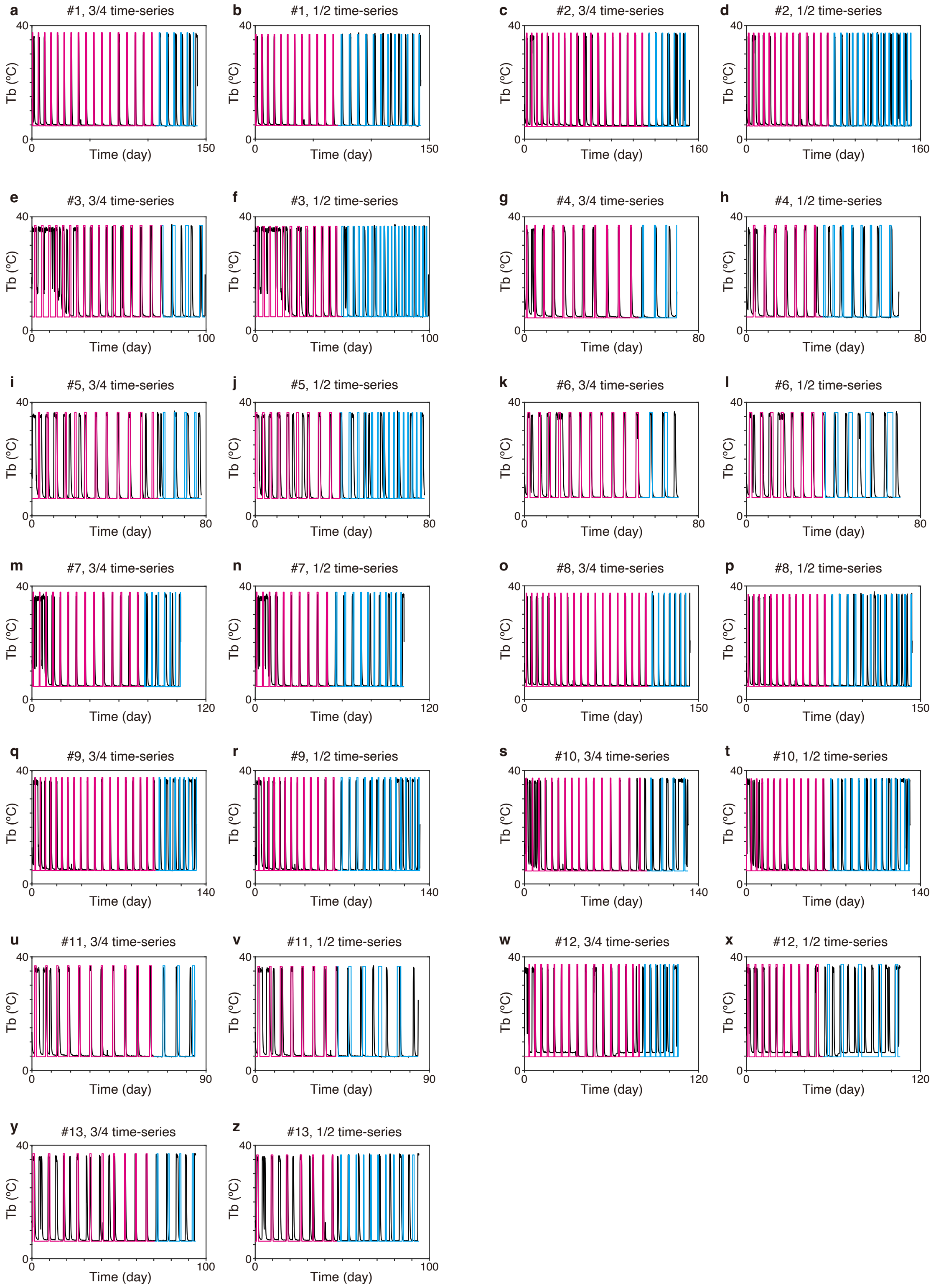

Supplementary Fig. 17: Forecasting Tb fluctuation from the three-quarters or the first half of the whole Tb time-series for 13 Syrian hamster individual data using AIC (a-z). Tb time-series was reconstructed (magenta) and predicted (cyan). Recorded Tb data is shown by black. Parameter ranges are shown in Supplementary Table 2.

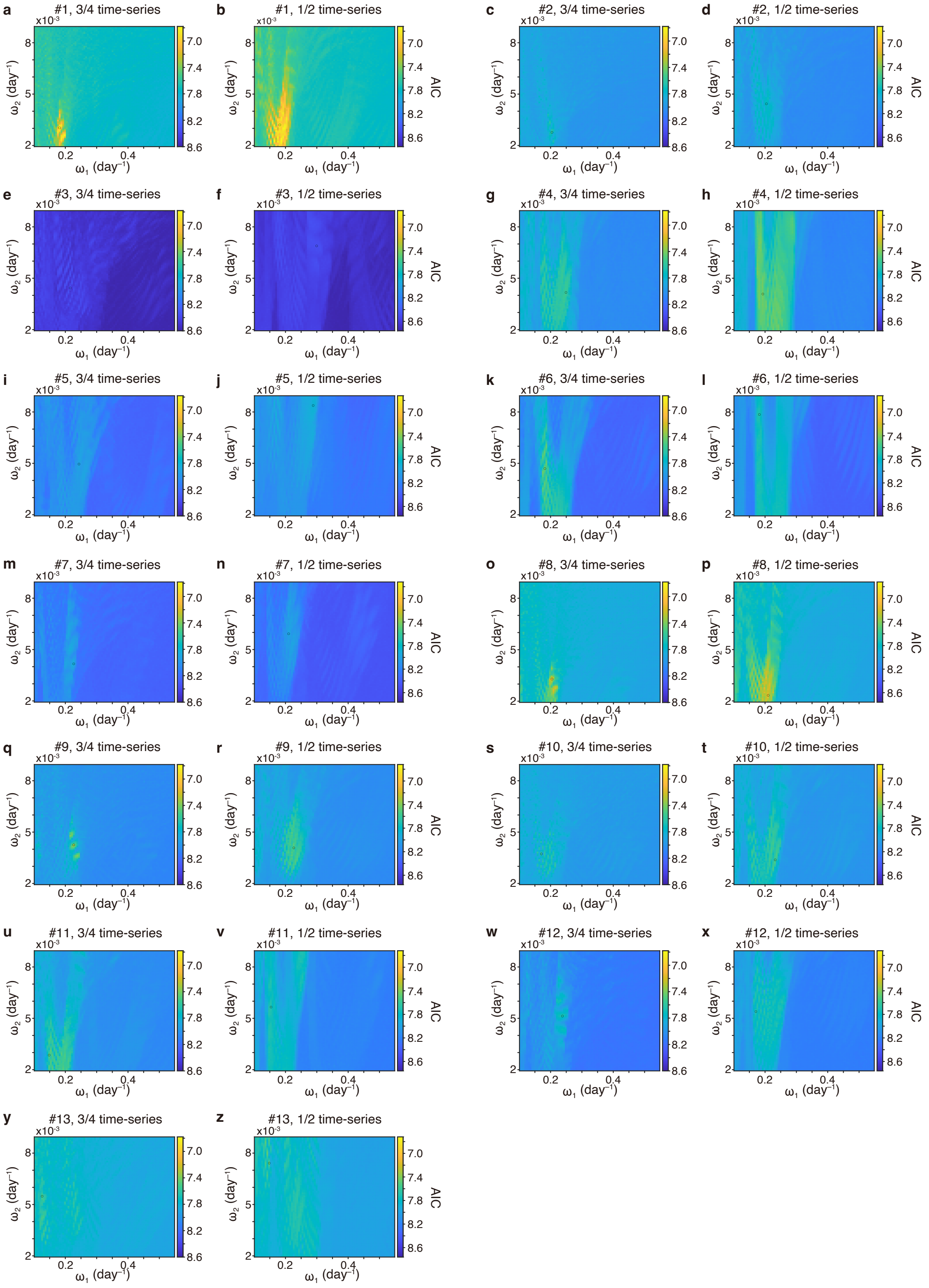

Supplementary Fig. 18: Distribution of AIC as a function of faster (ω_1) and slower (ω_2) frequencies of Syrian hamster estimated from the three-quarters or the first half of the whole Tb time-series (a-z). The best-fit parameter, yielding the minimum AIC (circle) was used in Supplementary Fig. 17. Parameter ranges are shown in Supplementary Table 2.

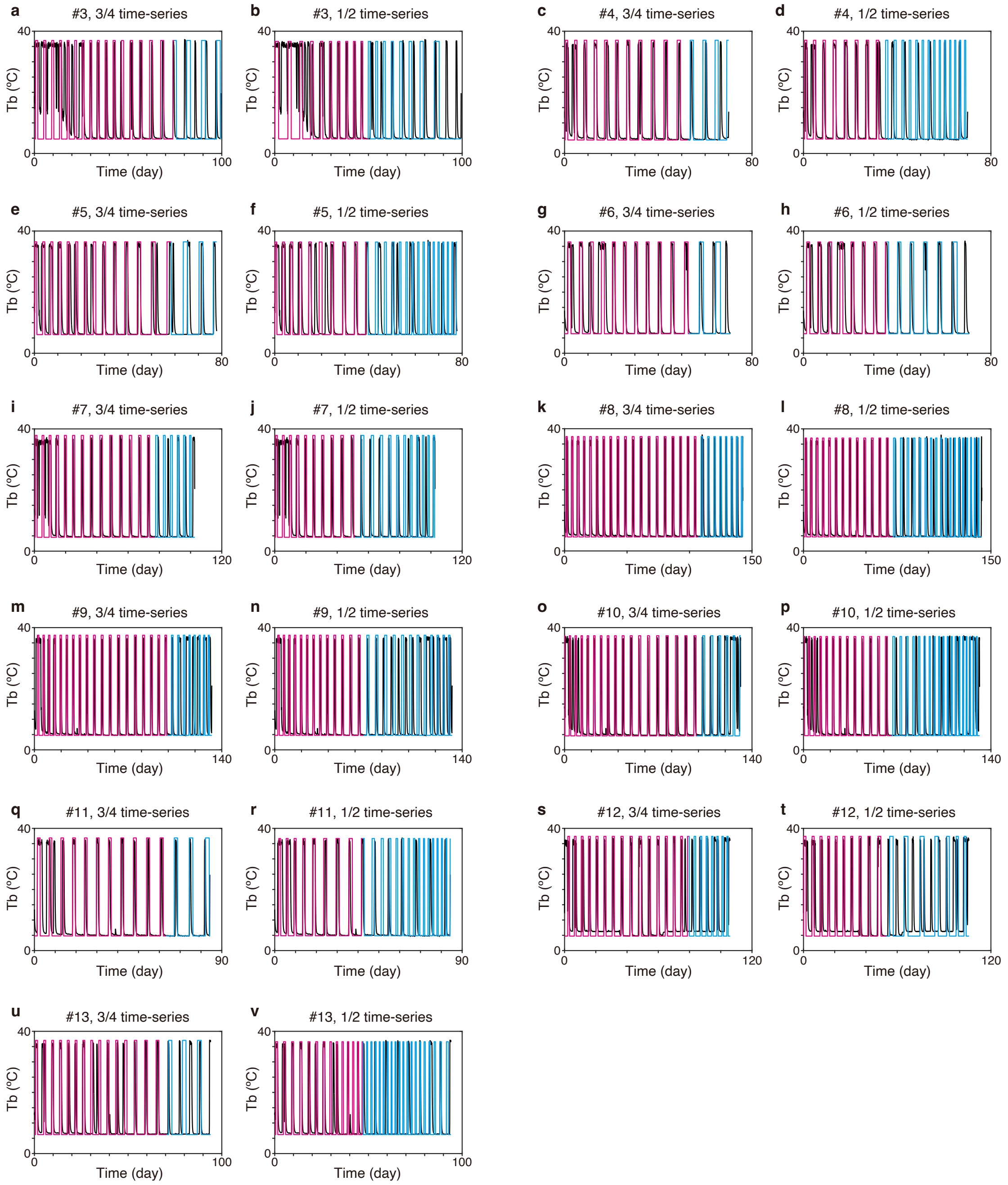

Supplementary Fig. 19: Forecasting Tb fluctuation of from the three-quarters or the first half of the whole Tb time-series for 11 Syrian hamster individual data using IS divergence (a-v). Tb time-series was reconstructed (magenta) and predicted (cyan). Recorded Tb data is shown by black. A total of 13 individual datasets, including 2 from Fig. 5a-d were analyzed. Parameter ranges are shown in Supplementary Table 2.

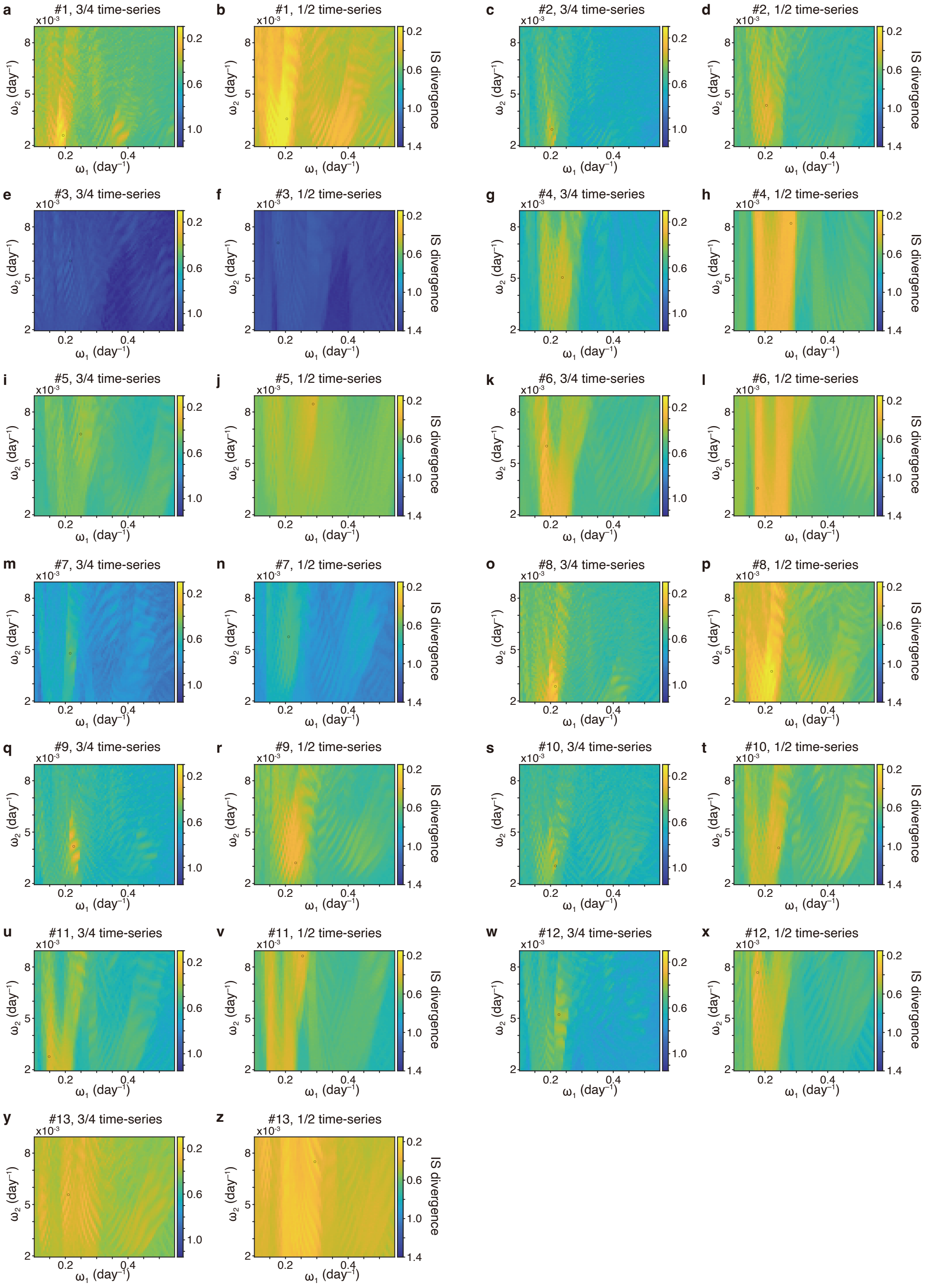

Supplementary Fig. 20: Distribution of IS divergence as a function of faster (ω_1) and slower (ω_2) frequency of Syrian hamster estimated from the three-quarters or the first half of the whole Tb time-series (a-z). The best parameter set, yielding the minimum IS divergence (circle) was used in Fig. 5a-d and Supplementary Fig. 19. Parameter ranges are shown in Supplementary Table 2.

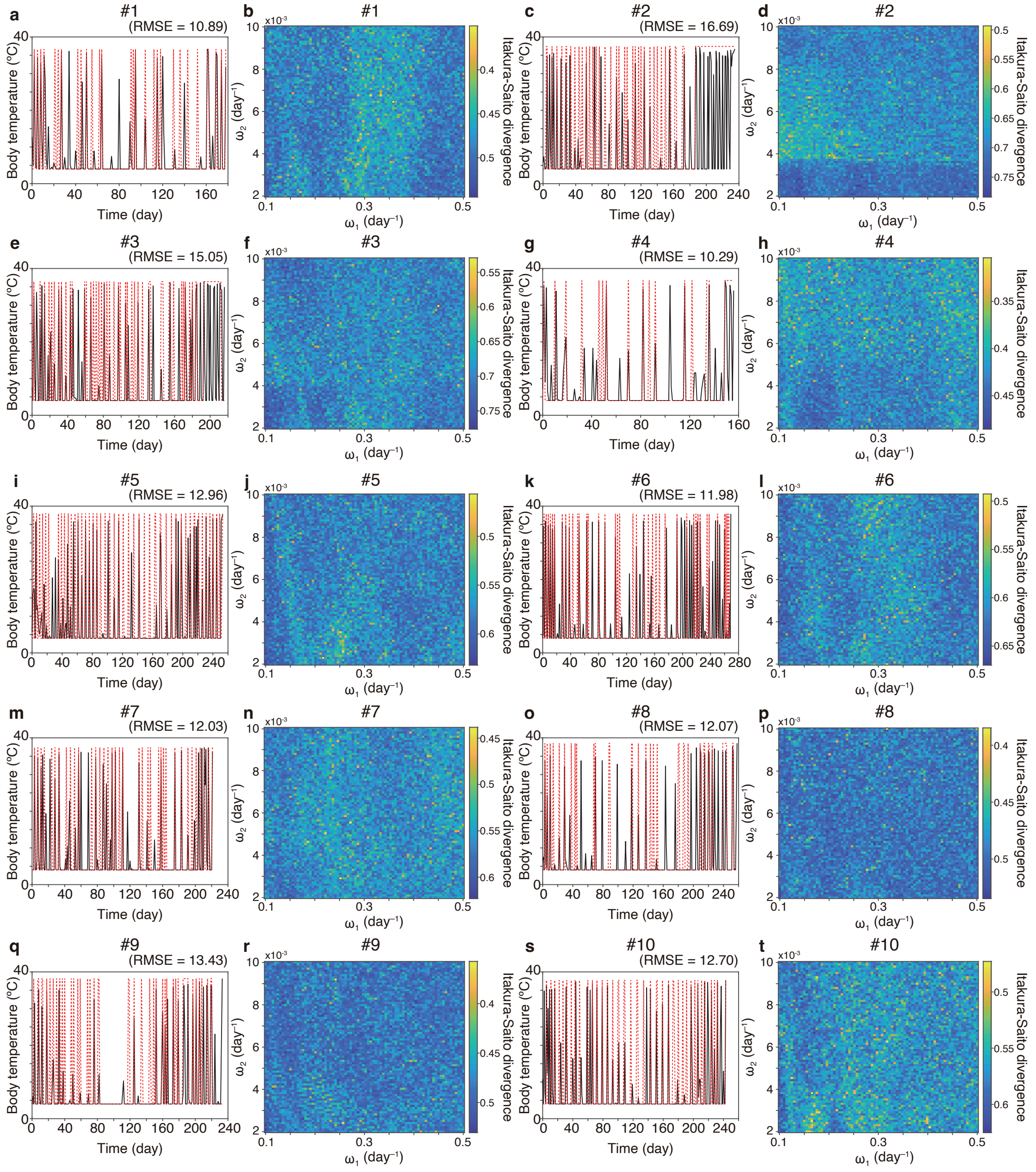

Supplementary Fig. 21: FM model simulation (red) with the best parameter set for Tb time series in 10 ground squirrels. The best parameter set was chosen using the minimum IS-divergence. RMSE represents the root mean squared error. Parameter ranges are shown in Supplementary Table 4.

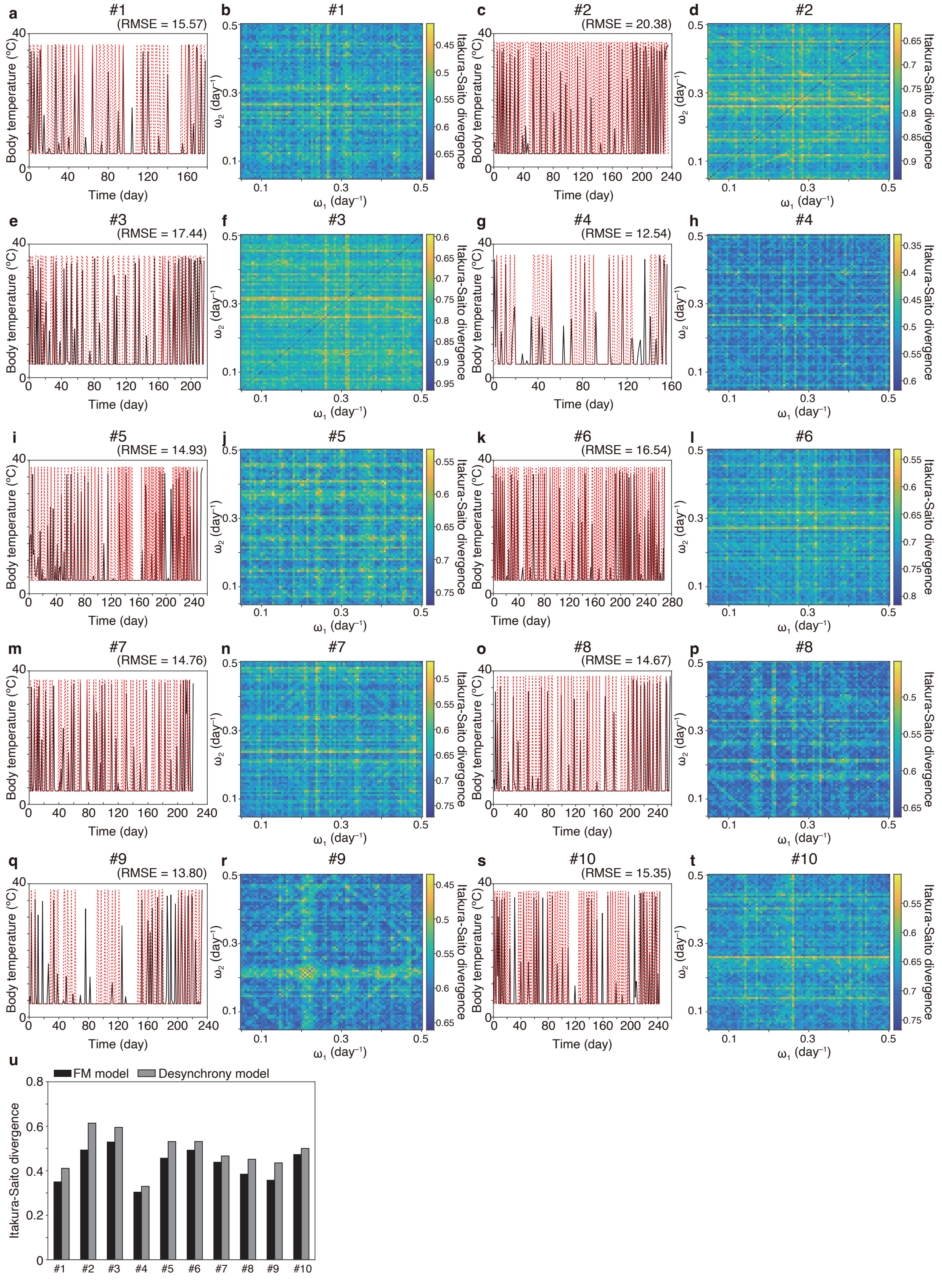

Supplementary Fig. 22: Desynchrony model simulation (red) with the best parameter set for Tb time series in 10 ground squirrels. Time series and distribution of IS values for desynchrony model (**a-t**). The best parameter set was chosen using the minimum IS-divergence. RMSE represents the root mean squared error. Parameter ranges are shown in Supplementary Table 5. (**u**) Comparison of the two models for realizing each individual time series (#1–10). The likelihood values of the FM (black) and desynchrony models (gray) were compared using IS divergence.

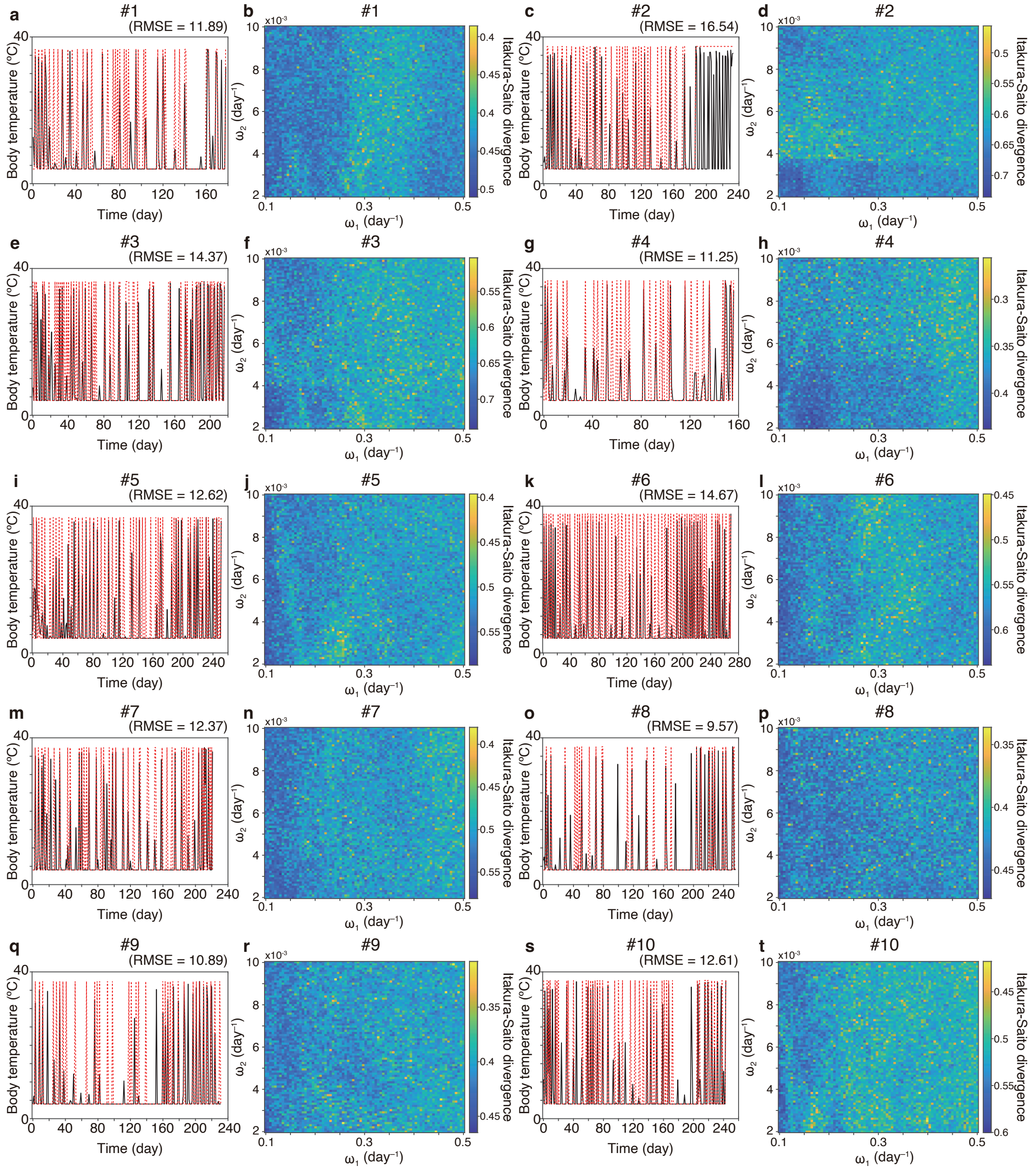

Supplementary Fig. 23: Simulation of the FM model (red) applied to Tb time series in 10 ground squirrels, including Gaussian noise and utilizing the best parameter set. The simulation was repeated 75 times to account for Gaussian noise. The best parameter set was chosen using the minimum IS-divergence across these 75 trials. RMSE represents the root mean squared error. Parameter ranges are shown in Supplementary Table 4.

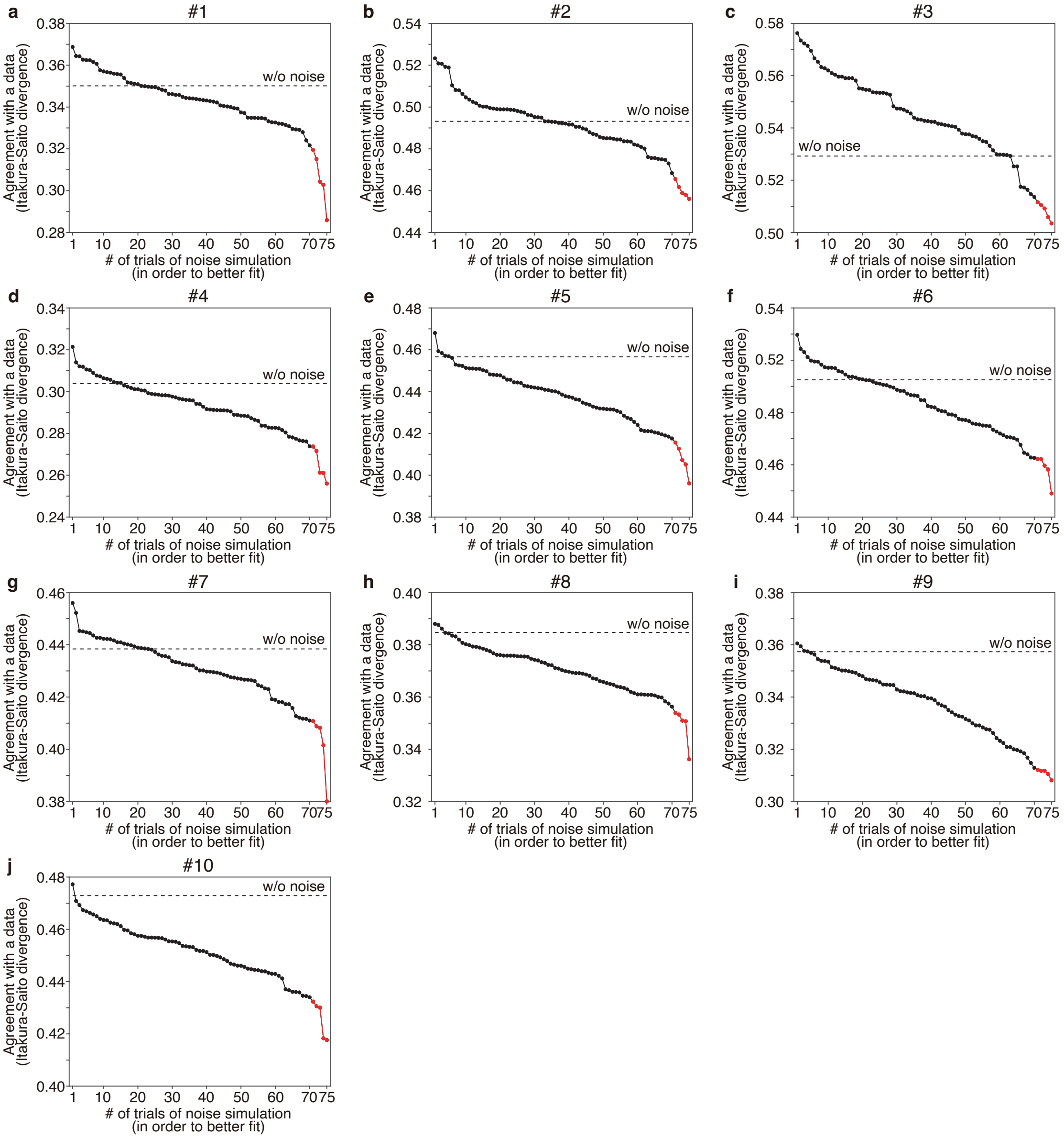

Supplementary Fig. 24: Comparison between the FM model simulation incorporating Gaussian noise and experimental Tb time series in 10 ground squirrels across 75 trials. The noise simulation was customized to fit the individual experimental data of the ground squirrels. The data were sorted in ascending order based on IS divergence values. The IS divergence value of the dotted line was for the best parameter set of the simulation when there was no noise (Supplementary Fig. 21). The average values of the longer period (ω_2) corresponding to the five lowest IS values (depicted in red) were utilized in Fig. 6e.

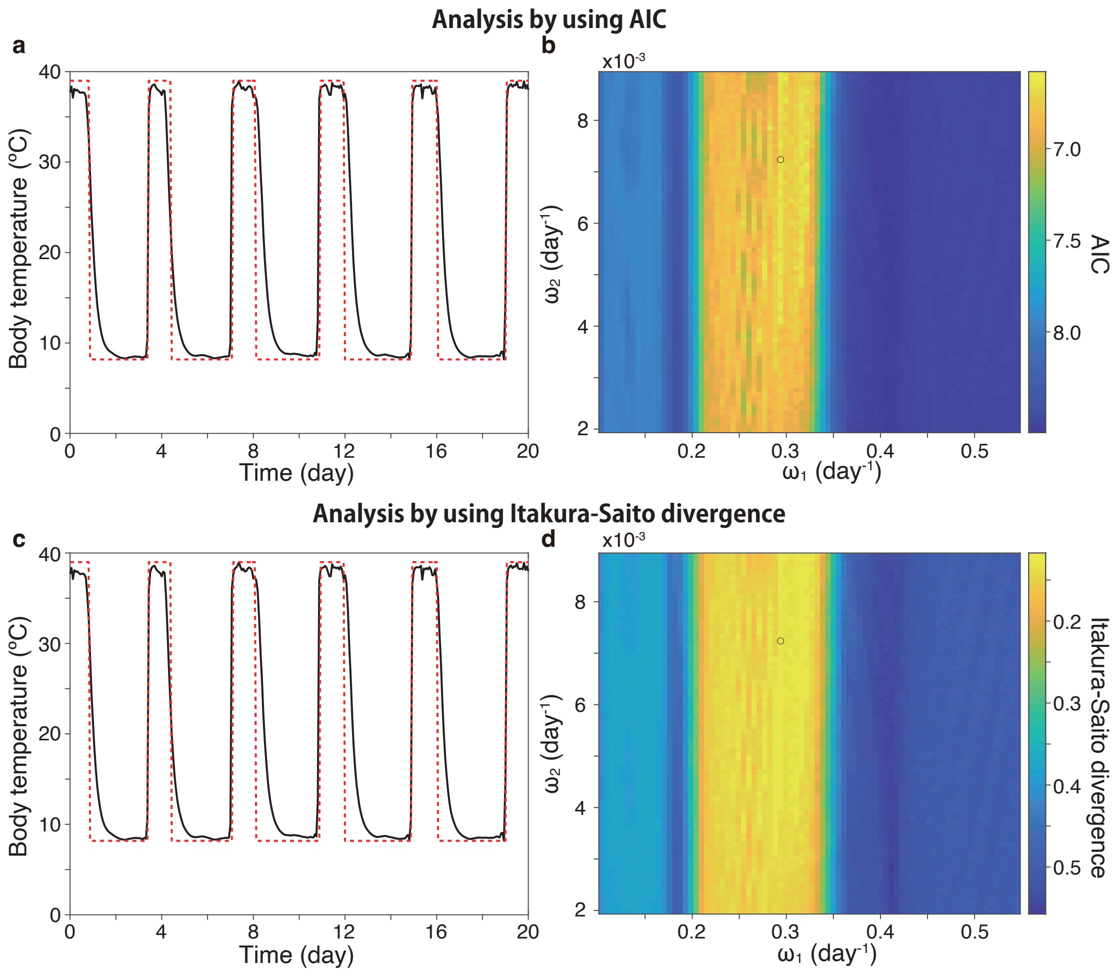

Supplementary Fig. 25: Simulation of the FM model (depicted in red) utilizing the best parameter set, based on Tb time series data published for European hamster. The time-series is sourced from Fig. 1A in Revel et al., 2007[45]. We conducted estimations using both the AIC (**a,b**) and IS divergence (**c,d**). The FM model's shorter and longer period, which minimize both AIC and IS divergence were 3.4 and 138 days respectively. Parameter ranges are shown in Supplementary Table 2.

Parameter	Lower limit	Upper limit	Number of intervals
ω_1	0.104 /days	1.02 /days	165
ω_2	0.00197 /days	0.00890 /days	80
ϕ_1	0	2π	20
ϕ_2	0	2π	20
A_2	0.124	0.206	20
θ	0.675	0.990	20

Supplementary Table 1: Parameter ranges for statistical analysis of FM model applied to Syrian hamster data. Upper and lower limits for parameter estimation were determined from preliminary estimation so that the model is likely to reproduce the torpor-arousal cycles of Syrian hamster in Fig. 2, 3, and Supplementary Fig. 6, 8, 11, 13.

Parameter	Lower limit	Upper limit	Number of intervals
ω_1	0.104 /days	0.546 /days	80
ω_2	0.00197 /days	0.00890 /days	80
ϕ_1	0	2π	20
ϕ_2	0	2π	20
A_2	0.124	0.206	20
θ	0.675	0.990	20

Supplementary Table 2: Parameter ranges for statistical analysis of FM model applied to Syrian and European hamster data. Upper and lower limits for parameter estimation were determined from preliminary estimation so that the model is likely to reproduce the torpor-arousal cycles of Syrian hamster in Fig. 5, and Supplementary Fig. 7, 9, 12, 17-20 and European hamster in Supplementary Fig. 25.

Parameter	Lower limit	Upper limit	Number of intervals
ω_1	0.0500 /days	2.00 /days	80
ω_2	0.0500 /days	2.00 /days	80
ϕ_1	0	2π	20
ϕ_2	0	2π	20
A_2	1	3	20
θ	0	θ_{smax}	20

Supplementary Table 3: Parameter ranges for statistical analysis of desynchrony model applied to Syrian hamster data. Parameter, θ is the threshold for step function S (see Methods for details). Desynchrony model can yield quasiperiodic oscillations with fluctuating amplitudes for certain parameter sets. Upper limit for the threshold, θ_{smax} was set to be the smallest local maximum, multiplied by 0.99 so that the small amplitudes of oscillations from desynchrony model were reflected in simulating Tb fluctuation.

Parameter	Lower limit	Upper limit	Number of intervals
ω_1	0.1 /days	0.5 /days	80
ω_2	0.002 /days	0.01 /days	80
ϕ_1	0	2π	20
ϕ_2	0	2π	20
A_2	0.1	0.2	20
θ	0.60	0.99	20

Supplementary Table 4: Parameter ranges for statistical analysis of FM model applied to 13-lined ground squirrel data. Upper and lower limits for parameter estimation were determined from preliminary estimation so that the model is likely to reproduce the torpor-arousal cycles of 13-lined ground squirrels.

Parameter	Lower limit	Upper limit	Number of intervals
ω_1	0.05 /days	0.5 /days	80
ω_2	0.05 /days	0.5 /days	80
ϕ_1	0	2π	20
ϕ_2	0	2π	20
A_2	1	3	20
θ	0	θ_{smax}	20

Supplementary Table 5: Parameter ranges for statistical analysis of desynchrony model applied to 13-lined ground squirrel data. Parameter, θ is the threshold for step function S (see Methods for details). Desynchrony model can yield quasiperiodic oscillations with fluctuating amplitudes for certain parameter sets. Upper limit for the threshold, θ_{smax} was set to be the smallest local maximum, multiplied by 0.99 so that the small amplitudes of oscillations from desynchrony model were reflected in simulating Tb fluctuation.

Dataset S1 (separate file). Original time-series of body temperature fluctuation during hibernation.
